# Two Novel Phenylpropanoid Trimers From *Ligusticum chuanxiong* Hort With Inhibitory Activities on Alpha-Hemolysin Secreted by *Staphylococcus aureus*

**DOI:** 10.3389/fchem.2022.877469

**Published:** 2022-03-30

**Authors:** Shi-Jie Wan, Han-Gui Ren, Jia-Ming Jiang, Gang Xu, Yu Xu, Si-Min Chen, Gan Chen, Dan Zheng, Man Yuan, Hong Zhang, Hong-Xi Xu

**Affiliations:** ^1^ School of Pharmacy, Shanghai University of Traditional Chinese Medicine, Shanghai, China; ^2^ Engineering Research Center of Shanghai Colleges for TCM New Drug Discovery, Shanghai, China; ^3^ State Key Laboratory of Phytochemistry and Plant Resources in West China and Yunnan Key Laboratory of Natural Medicinal Chemistry, Kunming Institute of Botany, Chinese Academy of Sciences, Kunming, China; ^4^ Center for Translational Medicine and Shanghai Key Laboratory of Diabetes Mellitus, Shanghai JiaoTong University Affiliated Sixth People’S Hospital, Shanghai, China; ^5^ Shuguang Hospital, Shanghai University of Traditional Chinese Medicine, Shanghai, China

**Keywords:** *Staphylococcus aureus*, alpha-hemolysin, *Ligusticum chuanxiong* Hort, lignan, anti-virulence

## Abstract

The emergence of antibiotic resistance in *Staphylococcus aureus* has necessitated the development of innovative anti-infective agents acting on novel targets. Alpha-hemolysin (Hla), a key virulence factor of *S. aureus*, is known to cause various cell damage and death. In this study, with bioassay-guided fractionation, a pair of unusual epimeric lignan trimers, ligustchuanes A and B (**1** and **2**), were isolated from the rhizomes of *Ligusticum chuanxiong* Hort, together with two known phthalides being identified by UPLC-QTOF-MS. To the best of our knowledge, trimers with rare C8-C9″-type neolignan and ferulic acid fragments have not been identified in any natural product. Both of them were isolated as racemic mixtures, and their absolute configurations were determined by comparing experimental and calculated ECD spectra after enantioseparation. Ligustchuane B exhibited an outstanding inhibitory effect on *α*-hemolysin expression in both MRSA USA300 LAC and MSSA Newman strains at concentrations of 3 and 6 *μ*M, respectively. Notably, a mouse model of infection further demonstrated that ligustchuane B could attenuate MRSA virulence *in vivo*.

## Introduction

*Staphylococcus aureus* is an ubiquitous human pathogen that causes a broad array of diseases, ranging from superficial infections to severe, life-threatening invasive infections such as endocarditis, septic arthritis, and sepsis (Gupta et al., 2013; Missiakas and Schneewind, 2016). The emergence and spread of multidrug-resistant strains with enhanced infectivity and virulence, such as the community-associated methicillin-resistant *S. aureus* (MRSA), along with a dearth of new antimicrobial agent discovery in recent decades, have exacerbated *S. aureus* infections (Kale and Dhawan, 2016). Moreover, *S. aureus* has evolved the ability to produce various virulence factors to destroy host tissue, overcome host’s immune response and multiply rapidly in the host (Tang et al., 2019). Given that most virulence factors are nonessential for bacterial survival, in principle, antimicrobial agents inhibiting microbial virulence without affecting its growth would potentially exert less selective pressure for the development of drug resistance (Abbas et al., 2020). Therefore, a new strategy focusing on anti-virulence therapy has emerged as a research priority in recent years (Sully et al., 2014; Xuewen et al., 2018; Parlet et al., 2019).

*α*-hemolysin (*α*-toxin, Hla), a major virulence-associated protein synthesized by *S. aureus,* forms heptameric transmembrane pores in target cell membranes (including erythrocytes, alveolar epithelial cells, lymphocytes, monocytes, and macrophages), leading to cell damage and death (Gupta et al., 2013; Parlet et al., 2019; Tang et al., 2019). Several studies have demonstrated the indispensable role of Hla in virulence of *S. aureus* (Berube and Bubeck Wardenburg, 2013; Berube et al., 2014; Duan et al., 2018; Smith et al., 2018). One of them reported that Hla-deficient mutants significantly reduced visceral injury in a mouse infection model, suggesting Hla as an ideal target for the development of anti-virulence drugs to combat *S. aureus* infection without affecting bacterial growth to cause drug resistance (Jensen et al., 2008; Montgomery et al., 2010; Hong et al., 2014). However, until now, small-molecule inhibitors targeting Hla are still lacking.

Natural products, especially traditional Chinese medicines (TCMs), are rich in bioactive components which present opportunities for the discovery of novel drug candidates (Yang et al., 2020). A library of TCM plant extracts was screened by western blotting analysis and hemolysis assay for their ability to inhibit Hla expression *in vitro*. Interestingly, the petroleum ether (PE)-soluble fraction of the 80% EtOH extract of the rhizomes of *Ligusticum chuanxiong* Hort (chuanxiong), named LCA, was found to be a promising hit, as it could inhibit the expression of Hla at a concentration of 200 *μ*g/mL against both methicillin-sensitive *S. aureus* (MSSA) and MRSA (Supplementary Figure S3). Moreover, the potential therapeutic effect of LCA was further demonstrated in a *S. aureus*-abdominal infection mouse model (Supplementary Figure S4). However, no visible effect on bacterial growth was discovered when applying LCA (400 *μ*g/mL) to coculture with *S. aureus* (Supplementary Figure S5). The minimal inhibitory concentration (MIC) values of LCA were later determined to be 800 *μ*g/mL against both strains, which indicated that LCA could attenuate the virulence of *S. aureus* by inhibiting Hla expression instead of killing it. This interesting phenomenon suggested that LCA may contain some secondary metabolites with anti-virulence activity against *S. aureus*, which is worthy of further study.

Chuanxiong, a traditional Chinese medicine first recorded in Shennong’s Herbal Classic of Materia Medica, is commonly used to treat headache, rheumatic arthralgia, menstrual disorders, post-traumatic swelling pain, and coronary heart diseases in China (Yan et al., 2008; Chen et al., 2018; Zhang et al., 2018; Zhang X et al., 2019). Previous pharmacological investigations have revealed that ligustilide and ferulic acid showed weak bacteriostatic activity against *S. aureus*. However, the anti-virulence effect of chuanxiong and its bioactive components have not been investigated (Borges et al., 2013; Pannek et al., 2018). In view of this, we performed a detailed phytochemical study to further explore the medicinal value of chuanxiong while looking for novel compounds possessing anti-virulence activity meanwhile.

## Materials and Methods

### General Experimental Procedures

Optical rotations were measured using an Autopol VI polarimeter. IR spectra were obtained from Perkin-Elmer 577 spectrometers. Ultraviolet absorption spectra were recorded in MeCN (25 *μ*g/mL) on a UV-2401 PC spectrophotometer. ECD spectra were recorded in MeCN (50 *μ*g/mL) on a Chirascan-plus spectrometer (Applied Photophysics Ltd., Surrey, United Kingdom). NMR spectra were measured in DMSO-*d*_*6*_ on Bruker AV-400 and Bruker AV-600 spectrometers and calibrated by the solvent peak used. Mass spectrometry was performed on a SYNAPT G2-Si HDMS (Waters Corp., Manchester, United Kingdom) with an electrospray ion source (Waters, Milford, MA) connected to a lock-mass apparatus, which performed real-time calibration correction. Column chromatography was performed with CHP20P MCI gel (75–150 *μ*m, Mitsubishi Chemical Corporation, Japan) and Sephadex LH-20 (GE Healthcare Bio-Sciences AB, Sweden). A Waters 2535 Series machine equipped with a X-bridge C_18_ column (4.6 × 250 mm, 5 *μ*m) was used for HPLC analysis, and a preparative X-bridge Prep C_18_ OBD column (19 × 250 mm, 5 *μ*m) was used for sample preparation. Enantioseparations were performed by a Waters Acquity UPC^2^ system (Waters, Milford, MA, United States) with a sample manager, binary solvent manager, compensation solvent pump and column manager on a Daicel Chiralpak IG column (5 *μ*m, 250 × 4.6 mm).

### Plant Material

The crude plant of chuanxiong was purchased from a Chinese herbal medicine market in Sichuan Province in China and identified by associate professor Hong-Mei Zhang at Shanghai University of Traditional Chinese Medicine as the rhizome of *Ligusticum chuanxiong* Hort. A voucher specimen (herbarium No. 20180501) was deposited at the School of Pharmacy, Shanghai University of Traditional Chinese Medicine.

### Extraction and Isolation

Air-dried and fragmented rhizomes of *Ligusticum chuanxiong* Hort (500 g) were soaked in 80% EtOH for 30 min at room temperature and then extracted by heat reflux (3 × 5 L). The obtained solutions were combined and concentrated in a rotary evaporator at 45°C to obtain the EtOH-soluble portion (149 g). The residue was suspended in H_2_O (300 mL) and extracted with PE (5 × 300 mL) to obtain the dried PE-soluble fraction named LCA (36 g), which was later applied to an MCI chromatography column and successively eluted with 50, 80 and 95% EtOH. The fraction eluted with 50% EtOH was named LCAI (25.8 g), while the fraction eluted with 80% EtOH was named LCAII (7.1 g). The last fraction eluted with 95% EtOH was named LCAIII (2.9 g). LCAII was then subjected to Sephadex LH-20 eluted with MeOH to yield subfraction LCAIIB (231 mg), which was later purified with preparative HPLC using a gradient of 45% MeCN-H_2_O (0.1% formic acid) at a flow rate of 20 mL/min and afforded ligustchuanes A (10.2 mg) and B (19.8 mg). As both compounds **1** and **2** were obtained as racemic mixtures, enantiomers (+) **1** (0.56 mg), (−) **1** (0.63 mg), (+) **2** (0.72 mg) and (−) **2** (0.77 mg) were separated via chiral-phase UPC^2^ on a Daicel Chiralpak IG column [CH_3_OH (A), CO_2_ (B); 20% A (0–27 min), flow rate of 2 mL/min; concentration of 50 mg/mL; 6 injections with 10 *μ*L]. Compound (+) **1** was eluted at 24.9 min, and compound (−) **1** was eluted at 15.7 min, while compound (+) **2** was eluted at 11.8 min and compound (−) **2** was eluted at 14.2 min.

### Spectroscopic Data

(+) Ligustchuane A (+) **1**: yellow oil; [*α*]^20^_D_ +4.0 (c 0.1, MeOH); (−) Ligustchuane A (−) **1**: yellow oil; [*α*]^20^_D_ −6.0 (c 0.1, MeOH); UV (MeCN) *λ*_max_ (log ε) 271 (3.98), 287 (3.96), 315 (3.92) nm; IR (KBr) *υ*_max_ 2972, 1695, 1596, 1511, 1452, 1429, 1372, 1267, 1156, 1120, 1030, 970, 849, 818 cm^−1^; ^1^H NMR data, see Table 1; ^13^C NMR data, see Table 1; HRESIMS *m/z* 563.2270 [M−H] ^−^ (calcd for C_32_H_35_O_9_, 563.2281).

**TABLE 1:** ^1^H and^13^C NMR data (DMSO-*d*_6_) for compounds 1^a^ and 2^b^.

pos.	1		2	
	*δ*_C_	*δ*_H_ (*J* in Hz)	*δ*_C_	*δ*_H_ (*J* in Hz)
1	131.4			
2	110.7	6.83, d (1.8)	110.7	6.85, d (1.8)
3	147.5		147.5	
4	145.9		145.9	
5	115.5	6.79, d (8.2)	115.5	6.65, d (8.1)
6	119.4	6.70, dd (8.2, 1.8)	119.8	6.73, dd (8.1, 1.8)
7	80.7	4.25, d (7.2)	80.7	4.21, d (7.3)
8	44.4	2.11, td (7.2, 4.0)	44.1	2.16, m
9	63.4	3.80, m, 3.99, dd (11.1, 4.0)	63.1	4.19, m, 4.26, m
10	63.5	3.26, m, 3.33, m	63.5	3.22, dd (9.5, 7.0), 3.29, m
11	15.2	1.11, t (7.0)	15.2	1.07, t (7.0)
1′	125.5		125.5	
2′	111.1	7.29, d (2.0)	111.1	7.30, d (2.0)
3′	147.9		147.9	
4′	149.4		149.4	
5′	115.2	6.79, d (8.2)	115.2	6.77, d (8.2)
6′	123.2	7.07, dd (8.2, 2.0)	123.2	7.07, dd (8.2, 2.0)
7′	144.9	7.48, d (15.9)	144.9	7.51, d (15.9)
8′	114.3	6.44, d (15.9)	114.3	6.46, d (15.9)
9′	166.5		166.7	
1″	128.9		128.9	
2″	109.4	6.94, d (2.0)	109.4	6.89, d (1.9)
3″	147.6		147.6	
4″	145.8		145.8	
5″	115.4	6.67, d (8.1)	115.4	6.79, d (8.2)
6″	119.0	6.74, dd (8.1, 2.0)	119.0	6.71, dd (8.2, 1.9)
7″	131.2	6.27, d (15.3)	131.3	6.17, d (15.1)
8″	125.2	6.08, dd (15.3, 7.6)	124.7	5.96, dt (15.1, 7.0)
9″	30.8	2.30, dt (14.9, 7.6), 2.48, m^c^	31.4	2.05, dt (14.6, 7.0), 2.14, m
MeO-3	55.5	3.74, s	55.6	3.75, s
MeO-3′	55.7	3.82, s	55.7	3.81, s
MeO-3″	55.4	3.74, s	55.5	3.72, s

a Recorded at 600 MHz (^1^H) and 150 MHz (^13^C).

b Recorded at 400 MHz (^1^H) and 100 MHz (^13^C).

c Overlapping with solvent peak.

(+) Ligustchuane B (+) **2**: yellow oil; [*α*]^25^_D_ +18.0 (c 0.06, MeOH); (−) Ligustchuane B (−) **2**: yellow oil; [*α*]^25^_D_ −16.7 (c 0.03, MeOH); UV (MeCN) *λ*_max_ (log ε) 270 (4.30), 287 (4.26), 315 (4.23) nm; IR (KBr) *υ*_max_ 2972, 1694, 1596, 1512, 1451, 1429, 1374, 1267, 1156, 1122, 1030, 968, 849, 817 cm^−1^; ^1^H NMR data, see Table 1; ^13^C NMR data, see Table 1; HRESIMS *m/z* 563.2292 [M−H] ^−^ (calcd for C_32_H_35_O_9_, 563.2281).

### Bacteria Strains and Reagents

The MSSA Newman strain and CA-MRSA USA300 LAC strain applied in the present study were cryopreserved at −80°C. Both strains were grown at 37°C in tryptic soy broth (TSB; OXOID CM0129) with gentle shaking in a shaker (TENSUC TS-100B Shaker Incubator) at 250 rpm and maintained on tryptic soy agar (TSA; OXOID CM0131) plates at 4°C. *Z*-ligustilide and senkyunolide A were purchased from the Chengdu Push Bio-Technology Co., Ltd. (Chengdu, China).

### Western Blotting Assay

LCA, LCAII and two compounds, *Z*-ligustilide and senkyunolide A, were dissolved in dimethyl sulfoxide (DMSO) to a concentration of 40 mg/mL, while compounds **1** and **2** were dissolved to a concentration of 9.6 mM to serve as stock solutions. An overnight culture of the *S. aureus* strain was transferred (1:100) into fresh TSB and incubated at 37°C when an OD_600_ of ∼0.3 was reached. The culture was then treated with the indicated concentrations of the fractions or compounds, incubated for another 3 h, and shaken at 250 rpm, while the blank culture was treated with 1% DMSO alone as control. A final DMSO concentration was kept at 1% (v/v). After centrifugation (10,000 g, 5 min), the supernatants were boiled with 5 × SDS–PAGE loading buffer, separated on 12.5% SDS–PAGE and transferred to nitrocellulose membranes (0.45 *μ*m, Merck Millipore). Ten percent dried skim milk was used to block the membranes for 1.5 h at room temperature, which were then stained by subsequent incubation with anti-*α*-toxin antibodies (polyclonal rabbit serum Sigma S7531; 1:10,000) overnight at 4°C and secondary antibodies (peroxidase-conjugated AffiniPure goat anti-rabbit IgG; ZSGB-BIO; 1:4000) at room temperature for 1.5 h. Protein expression was detected by chemiluminescent reaction and analyzed via a luminescent image analyzer (GE ImageQuant LAS 4000 min).

### Hemolysis Assay

Bacterial culture supernatant samples, prepared as described in the western blotting assay section, were applied for the hemolysis assay. One hundred microliters of the supernatant of each sample was incubated with 900 *μ*L of washed 1% rabbit erythrocytes for 20 min at 37°C. The culture supernatant from untreated cells served as a positive control (100% hemolysis). Following centrifugation (2000 g, 1 min), the OD_543_ of supernatant fluid was determined. The hemolysis percentage was then calculated after comparison with the control.

### Determination of the MIC of LCA

The minimal inhibitory concentration (MIC) of LCA against *S. aureus* strains was examined in triplicate using the standard microdilution method recommended by the Clinical and Laboratory Standards Institute.

### Molecular Docking Studies

The interaction mechanisms of compound (±) **2** on *α*-hemolysin heptamer were investigated with the AutoDock Vina1.1.2 program (Pagadala et al., 2017). The 3D crystal structure of the target protein of *α*-hemolysin was accessed through the protein data bank website (http://www.rcsb.org/pdb) (PDB ID: 6U49). From the crystal structure, water and ion molecules were removed, and appropriate hydrogen atoms were added under physiological pH conditions (pH = 7) using AutodockTools-1.5.6 software. In docking, the protein was considered rigid, while the ligands were flexible. To perform suitable docking for each ligand, we set the search space box parameters to 47.3–47.3–47.3 Å (direction x, y, and z) centered at (−10.1, 13.6, and 15.0) Å.

Based on the binding energy (∆G) results, the final docked conformations were ranked. The most favorable binding conformations had the lowest free energies, which were selected as suitable poses of binding and were later analyzed visually. Hydrogen bonding and hydrophobic interactions between ligands and proteins were visualized using PyMOL2.3.0 and LIGPLOT V 2.2.4 software (Yuan et al., 2017).

### Mouse Models of Intraperitoneal Infection

The experiments were performed according to our previous study (Zhang H et al., 2019). The overnight liquid cultures of *S. aureus* were diluted 20-fold in fresh TSB in a 50 mL flask and then cultured at 37°C for approximately 3 h to an OD_600_ of 0.6. The bacteria were later washed twice with phosphate-buffered saline (PBS) after being harvested. After weighing, 1 × 10^8^ CFU of the MSSA Newman strain suspended in PBS was administered to each female BALB/c mouse (18–20 g) (Beijing Vital River Laboratory Animal Technology Co., Ltd.) via intraperitoneal injection, recorded as day 0. From day −1 to day 2, these mice received intraperitoneal injections of LCA (100 mg/kg/d, once daily) or saline containing 20% PEG400 and 1% Tween 80 (mock). The animals were euthanized in day 3. The kidneys, spleens, hearts and livers were aseptically removed, recorded by photos, homogenized in PBS with 0.1% Triton X-100 to obtain single-cell suspensions, and quantified by the plating method.

With regard to MRSA strain USA300 LAC, all the procedures were similar to those of Newman, except 7.4 × 10^8^ CFU and 6.1 × 10^8^ CFU of bacteria were used for the infection model and intraperitoneal injections of LCAII and compound **2**, rather that LCA, were given once daily at 100 mg/kg/d and 10 mg/kg/d, respectively.

### Ethical Approval

All mouse experiments were performed according to institutional guidelines approved by the Committee for Animal Experiments, Shanghai University of Traditional Chinese Medicine, which are in accordance with guidelines for the ethical review of laboratory animal welfare approved by the General Administration of Quality Supervision, Inspection and Quarantine of the People’s Republic of China (2-6-2018). The animal study protocols were reviewed and approved by the Institutional Animal Care and Use Committee (IACUC) of the Shanghai Public Health Clinical Center (2019A00201).

### Statistical Analysis

Data were presented as the mean ± standard error (SE). Differences between two datasets were measured using the two-tailed Student’s *t* test and the nonparametric Mann–Whitney test (two-tailed). One-way ANOVA followed by Dunnett’s multiple comparison test was carried out to compare three or more groups using GraphPad Prism 6 software (GraphPad Software, Inc., San Diego, CA, United States). *p* values of <0.05 were considered statistically significant (*p < 0.05; **p < 0.01 and ***p < 0.001).

## Results

### Bioassay-Guided Fractionation

In follow-up work, LCA was further investigated with bioassay-guided fractionation. LCAII, one of the subfractions of LCA, showed stronger antihemolytic activity at a concentration of 50 *μ*g/mL against MSSA Newman and 25 *μ*g/mL against the MRSA USA300 LAC strain without exhibiting bacteriostatic activity against bacteria (Supplementary Figures S5−S7). Furthermore, intraperitoneal treatment with LCAII led to a significant decrease in MRSA abundance in the visceral organs and weight loss of infected mice compared to the control group, showing significant anti-virulence activity against MRSA *in vivo* (Supplementary Figure S8). However, *Z*-ligustilide and senkyunolide A, two main components in LCAII identified by HRESIMS, showed weak activity against Hla expression (Supplementary Figures S5, S10, S11), indicating that the two components were not the main compounds accounting for the anti-virulence activity of LCAII.

**FIGURE 1:**
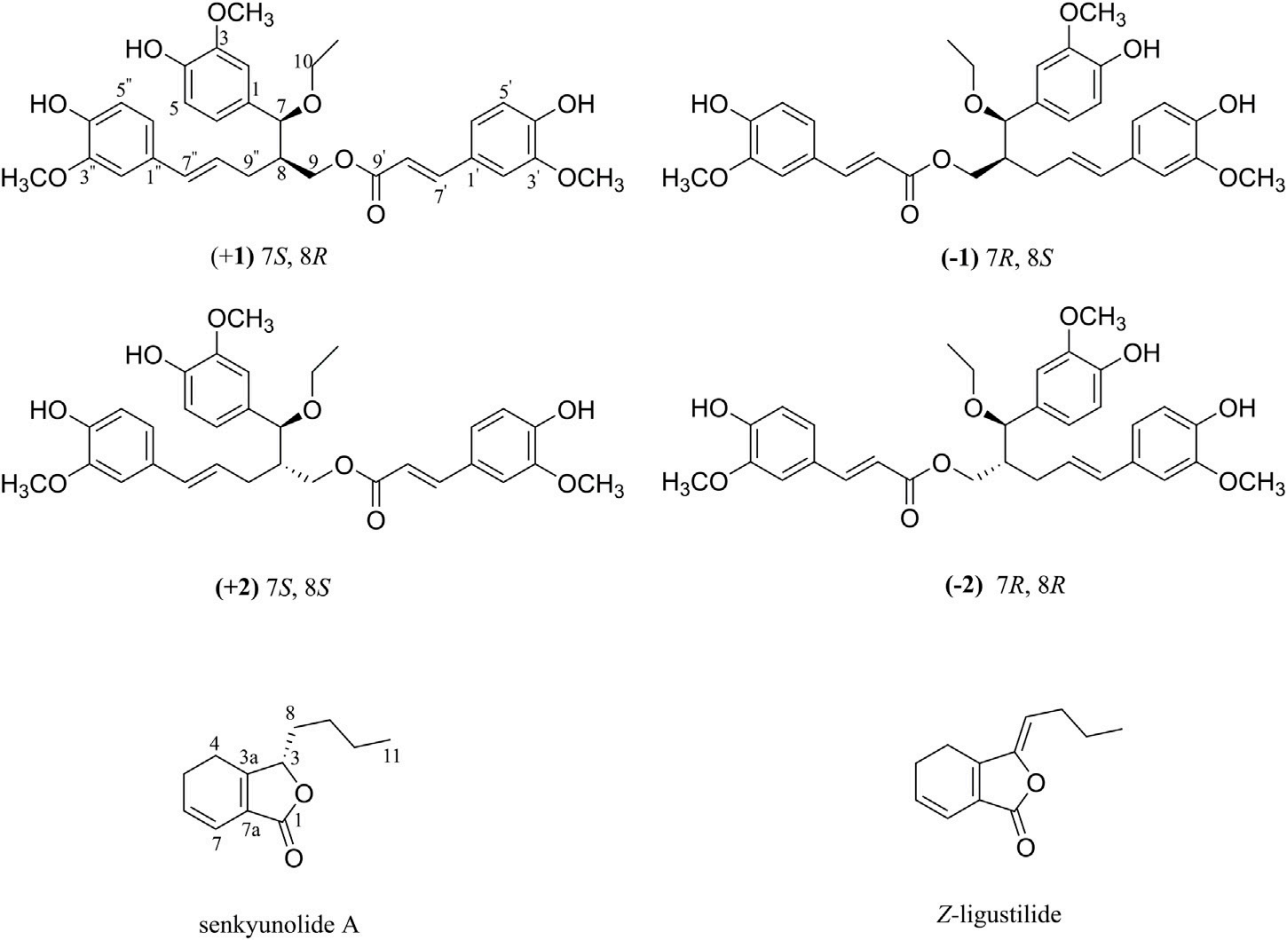
Chemical structures of (±) **1**, (±) **2**, senkyunolide A and *Z*-ligustilide.

For these reasons, with further bioactivity-guided fractionation of LCAII, LCAIIB (the subfraction of LCAII) showed stronger inhibition of Hla expression against *S. aureus* at a concentration of 12.5 *μ*g/mL (Supplementary Figure S12). Two rare lignan trimers (ligustchuanes A and B) were then isolated from LCAIIB by preparative HPLC (Figure 1). Their structures were assigned by 1D and 2D NMR spectroscopic analysis and mass spectrometry. Their absolute configurations were confirmed by comparing experimental and calculated ECD data.

### Structural Identification

Ligustchuane A (LCEA, **1**) was obtained as a yellow oil, and its molecular formula was designated C_32_H_36_O_9_ according to the pseudomolecular ion peak at *m/z* 563.2270 [M−H] ^−^ (calcd for C_32_H_35_O_9_, 563.2281) in its HRESIMS. The ^1^H NMR spectroscopic data of 1 (Table 1) exhibited the presence of three 1,3,4-trisubstituted benzene rings [*δ*_H_ 6.83 (1H, d, *J* = 1.8 Hz, H-2), 6.79 (1H, d, *J* = 8.2 Hz, H-5), 6.70 (1H, dd, *J* = 8.2, 1.8 Hz, H-6), 7.29 (1H, d, *J* = 2.0 Hz, H-2′), 6.79 (1H, d, *J* = 8.2 Hz, H-5′), 7.07 (1H, dd, *J* = 8.2, 2.0 Hz, H-6′), 6.94 (1H, d, *J* = 2.0 Hz, H-2″), 6.67 (1H, d, *J* = 8.1 Hz, H-5″), and 6.74 (1H, dd, *J* = 8.1, 2.0 Hz, H-6″)], four olefinic protons [*δ*_H_ 7.48 (1H, d, *J* = 15.9 Hz, H-7′), 6.44 (1H, d, *J* = 15.9 Hz, H-8′), 6.27 (1H, d, *J* = 15.3 Hz, H-7″), and 6.08 (1H, d, *J* = 15.3, 7.6 Hz, H-8″)], three methoxyl groups [*δ*_H_ 3.74 (3H, s, MeO-3), 3.82 (3H, s, MeO-3′), and 3.74 (3H, s, MeO-3″)] and one methyl singlet [*δ*_H_ 1.11 (1H, t, *J* = 7.0 Hz, H-11)]. Further analyses using ^13^C NMR, DEPT and HSQC (Supplementary Figures S21–S23) indicated the presence of 33 carbon signals, of which 12 could be assigned to a ferulic acid ester moiety (*δ*_C_ 125.5, C-1′; *δ*_C_ 111.1, C-2′; *δ*_C_ 147.9, C-3′; *δ*_C_ 149.4, C-4′; *δ*_C_ 115.2, C-5′; *δ*_C_ 123.2, C-6′; *δ*_C_ 144.9, C-7′; *δ*_C_ 114.3, C-8′; *δ*_C_ 166.5, C-9′; and *δ*_C_ 55.7, MeO-3′) and an ethoxyl group (*δ*_C_ 63.5, C-10 and *δ*_C_ 15.2, C-11). The remaining carbon signals included two C_6_-C_3_ units, indicating the presence of a lignan dimer. Thus, **1** was undoubtedly composed of the three moieties mentioned above.

**FIGURE 2:**
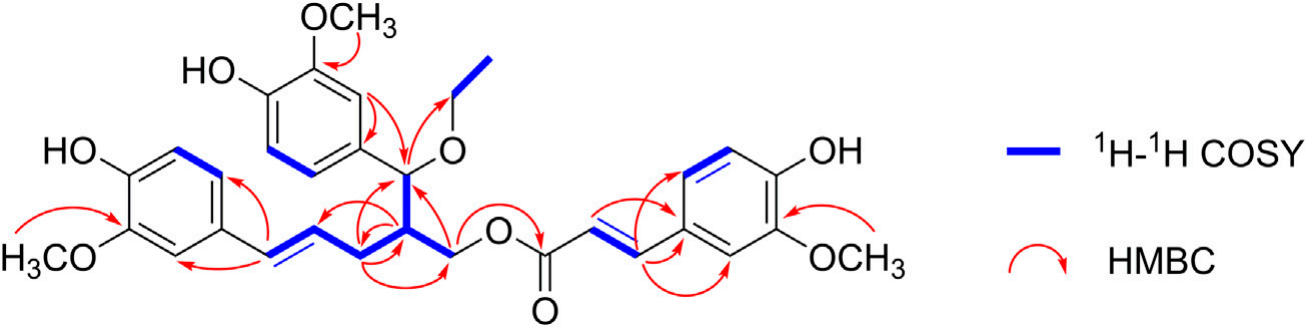
Selected ^1^H−^1^H COSY and ^1^H−^13^C HMBC correlations of (±) **1** and (±) **2**.

The planar structure of **1** was further determined using 2D NMR spectroscopy. The two C_6_-C_3_ units were linked by a bond between C-8 and C-9″ according to the HMBC correlations from H-9″ to C-7/C-8/C-9 and H-8 to C-8″/C-9″. Moreover, the location of the ethoxy group at C-7 and the ferulic acid ester moiety at C-9 were confirmed using HMBC correlations from H-7 to C-10 and H-9 to C-9′. Other key HMBC and ^1^H−^1^H COSY correlations are shown in Figure 2. Thus, **1** was an unprecedented lignan trimer condensed with C8-C9″-type neolignan, ferulic acid and ethoxyl groups.

**FIGURE 3:**
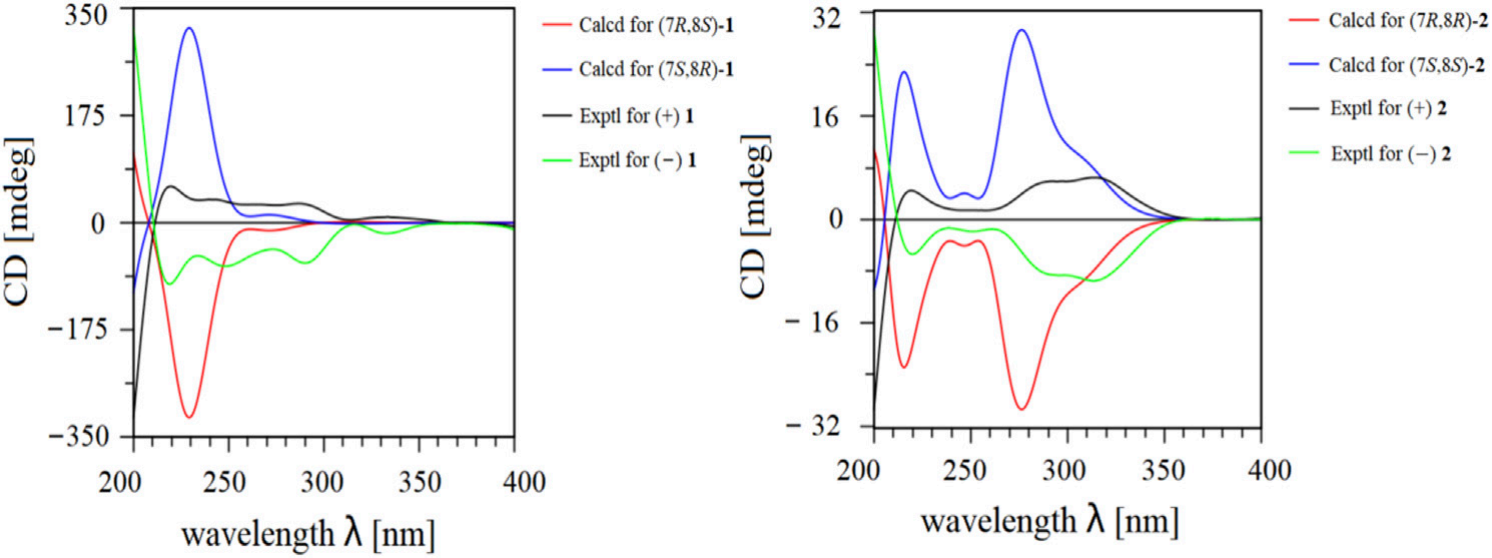
Experimental and calculated ECD data of (±) **1** and (±) **2**.

Given its flexible structure, the relative configurations at the chiral centers C-7 and C-8 could not be determined on the basis of NOESY data. Additionally, due to its oily texture, a suitable crystal was hard to acquire. Therefore, the absolute configuration of **1** was determined by comparison of the experimentally measured electronic circular dichroism (ECD) curves with the TDDFT-predicted curves. However, the measured ECD spectrum had no obvious cotton effect coupled with its specific rotation**,** [*α*]^20^_D_ -1.5 (c 1.0, MeOH), hinting at a racemic mixture (Supplementary Figure S18). Semi-preparative enantioseparation was subsequently achieved by chiral-phase UPC^2^ using a Daicel Chiralpak IG column to yield the two enantiomers, (+) **1** ([*α*]^20^_D_ + 4.0, t_R_ = 24.9 min) and (−) **1** ([*α*]^20^_D_ −6.0, t_R_ = 15.7 min) (Supplementary Figure S27). The specific rotation with opposite signs and mirror-image ECD curves also supported their enantiomeric relationship. Given the two chiral carbons in the structure, a total of four possible stereoisomers were deduced: (7*S*,8*S*)-**1**, (7*R*,8*S*)-**1**, (7*R*,8*R*)-**1**, and (7*S*,8*R*)-**1**. The calculated ECD spectrum of (7*S*,8*R*)-**1** agreed well with the experimental ECD spectrum of (+) **1**, while the other showed similar Cotton effects to (7*R*,8*S*)-**1** (Figure 3). Therefore, the absolute configurations of (+) **1** and (−) **1** were defined as (7*S*,8*R*) and (7*R*,8*S*), respectively.

Ligustchuane B (LCEB, **2**) was also isolated as a yellow oil. The molecular formula was determined to be C_32_H_36_O_9_ on the basis of a pseudomolecular ion [M−H] ^−^ in the HRESIMS at *m/z* 563.2292, which indicated that **2** is one of the isomers of **1**. In addition, ^1^H and ^13^C NMR spectra (Table 1) also revealed high similarities with those of **1**. 2D NMR spectroscopy (Supplementary Figures S34–S37) further confirmed the differences in the relative configuration between **1** and **2**. The NOE correlation of H-7/H-9/H-9″ in **1** could not correspondingly be found in **2**, suggesting that they were a pair of epimers. Similarly, **2** was also a racemic mixture due to its experimental ECD spectrum and specific rotation**,** [*α*]^20^_D_ +3.8 (c 1.0, MeOH) (Supplementary Figure S29). Two enantiomers were subsequently obtained by semipreparative enantioseparation as (+) **2** ([*α*]^25^_D_ +18.0, t_R_ = 11.8 min) and (−) **2** ([*α*]^25^_D_ −16.7, t_R_ = 14.2 min) (Supplementary Figure S39). By comparison of the experimental and calculated ECD curves, the absolute configurations of (+) **2** and (−) **2** were established as (7*S*,8*S*) and (7*R*,8*R*), respectively (Figure 3). To date, absolute configurations of all four stereoisomers have been presented as depicted.

However, the ethoxyl substituent groups were not common in the natural products, which might originate from the extract solution and isolation procedures. To determine whether **1** and **2** are artifacts, another sample of chuanxiong was extracted with 80% MeOH and subsequent fractionation and analytical processes were carried out in the absence of EtOH (Supplementary Material). As shown in Supplementary Figure S13, both compounds **1** and **2** could be detected in the new crude extract by UPLC-ESI-QTOF-MS. Thus, both trimers are natural products instead of extraction artifacts.

### Inhibitory Effects of *S. aureus* on Hla *in vitro*

**FIGURE 4:**
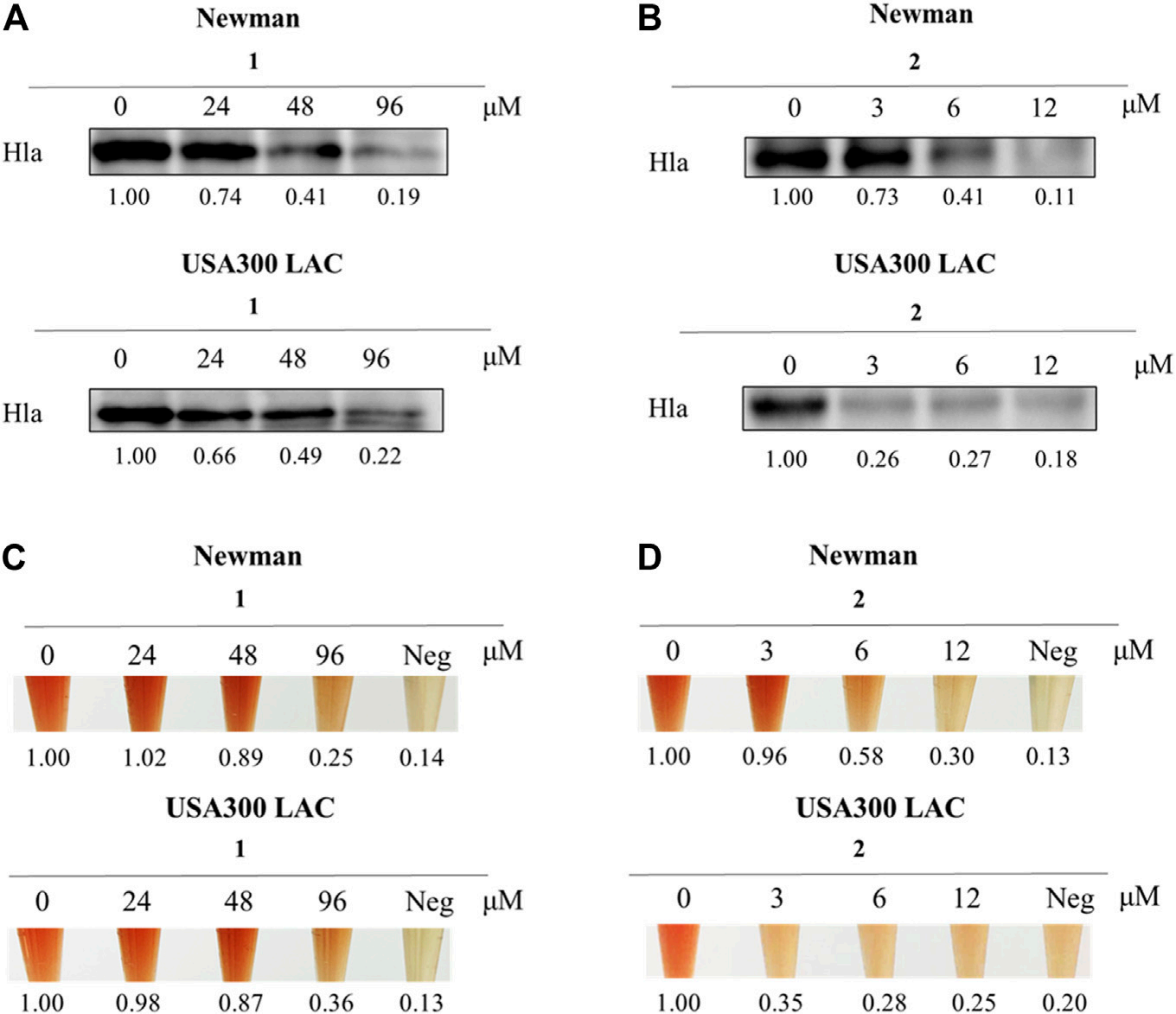
Compound **2** (LCEB) showed outstanding inhibition against Hla in both Newman and USA300 LAC strains. **(A)** Western blotting analysis of Hla expression in Newman and USA300 LAC strains treated with different concentrations of **1** (LCEA). **(B)** Western blotting analysis of Hla expression in Newman and USA300 LAC strains treated with different concentrations of LCEB. **(C)** Hemolysis assay in Newman and USA300 LAC strains treated with different concentrations of LCEA. **(D)** Hemolysis assay in Newman and USA300 LAC strains treated with different concentrations of LCEB.

Due to the tiny quantities of four enantiomers obtained from enantioseparation, both western blotting analysis and hemolysis assay were conducted on the racemic mixture of both compounds to evaluate the effect on Hla expression against *S. aureus*. Interestingly, as shown in Figure 4, the inhibitory effect of ligustchuane B on Hla expression against Newman was observed at a concentration of 6 *μ*M, while the corresponding inhibitory concentration of ligustchuane A was 96 *μ*M. Moreover, ligustchuane B showed a stronger inhibitory effect at a concentration of 3 *μ*M against USA300 LAC, with an effective concentration of ligustchuane A of 96 *μ*M. A hemolysis assay further confirmed the significant antihemolytic activity of ligustchuane B. However, *α*-hemolysin has been researched as a major target of *S. aureus* infection therapy for only a few years. So far, only one monoclonal antibody AR-301 has recently entered clinical trials, which led to no suitable small-molecule *α*-hemolysin inhibitor being able to serve as the positive control (Wang et al., 2016a; Wang et al., 2016b; Chen et al., 2016). But according to the previous studies (Lee et al., 2012; Zheng et al., 2021), ligustchuane B could be considered as one of the most potential small-molecule *α*-hemolysin inhibitors so far. More importantly, given the high structural similarity between ligustchuanes A and B but an approximately 32-fold difference in inhibitory concentration, the spatial configuration of chiral carbons C-7 and C-8 inevitably played an intensively important role in their anti-virulence activity against *S. aureus*.

### Anti-Virulence Effect of Ligustchuane B in the MRSA-Infected Mouse Model

**FIGURE 5:**
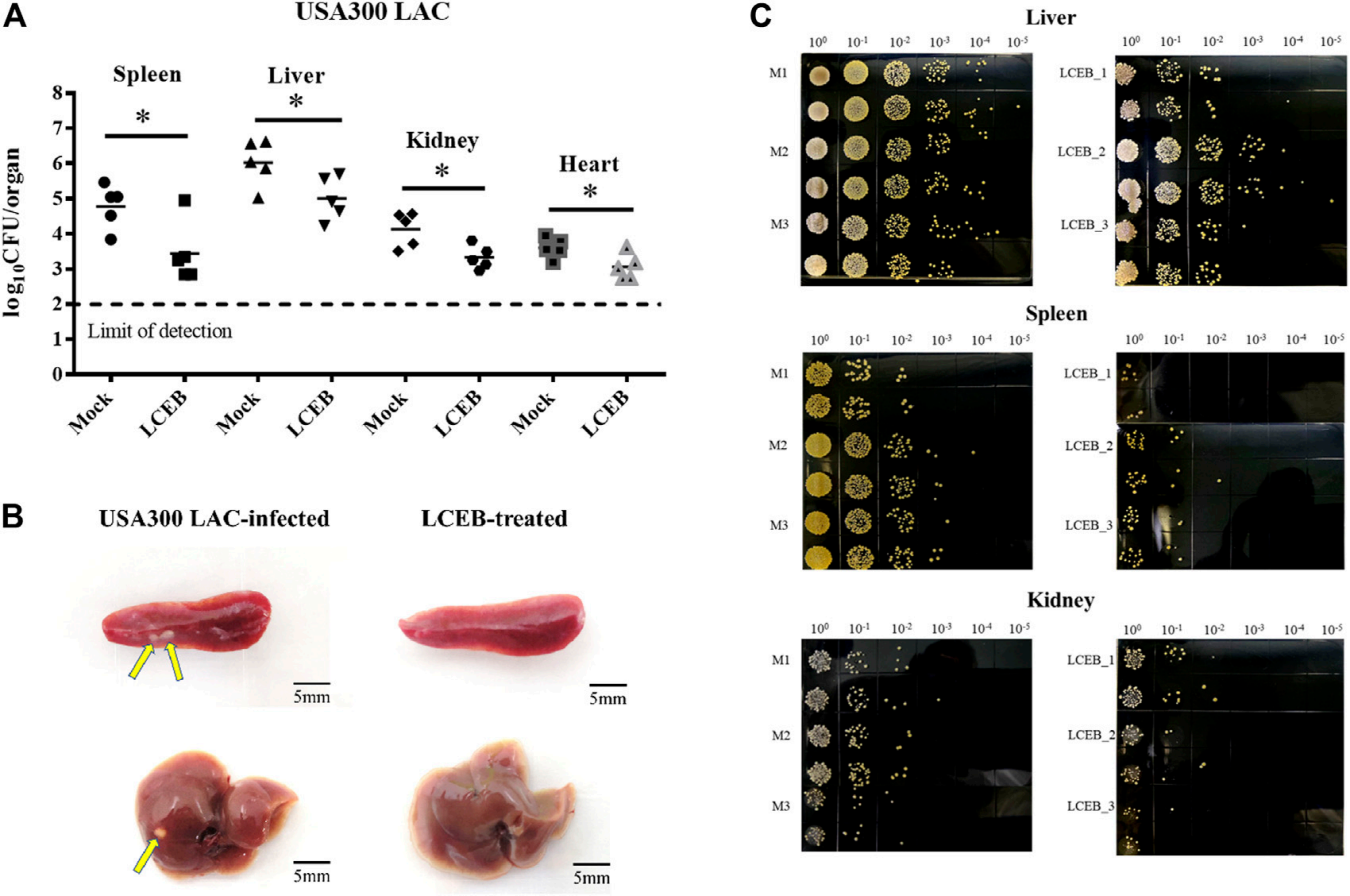
Effect of **2** (LCEB, 10 mg/kg/d) on the survival of the *S. aureus* USA300 LAC strain in the spleens, livers, kidneys and hearts of mice (*n* = 5) intraperitoneally challenged with 6.1 × 10^8^ CFU of bacteria. **(A)** Statistical analysis for the enumeration of colony forming units (CFU) is displayed. *p < 0.05 in comparison with control, Mann-Whitney test, two-tailed. Each symbol represents the value for an individual mouse. Horizontal bars indicate the observational means and the dashed line marks limit of detection. **(B)** Representative photographs of USA300 LAC-infected mice spleens and livers treated with or without LCEB. **(C)** Representative photographs of the TSA plates for CFU enumeration of the mice livers, spleens and kidneys infected by USA300 LAC with or without treatment of LCEB.

To further examine the role of ligustchuane B in reducing the virulence of MRSA *in vivo*, the USA300 LAC-infected mouse model was then applied. As expected, after the intraperitoneal injection of ligustchuane B, a significant decrease in bacterial burden was found in the spleen (∼1.34 log_10_ CFU/organ reduction), liver (∼1.01 log_10_ CFU/organ reduction), kidney (∼0.81 log_10_ CFU/organ reduction) and heart (∼0.54 log_10_ CFU/organ reduction) of the infected mice. Furthermore, fewer abscesses were observed in the visceral organs of infected mice treated with ligustchuane B (Figure 5). Although antibiotics are usually considered as the positive control on infective diseases, they treat them by directly killing bacteria instead of inhibiting virulence factors, rapidly leading to the drug resistance. Thus, antibiotics were not suitable to serve as the positive control in this animal experiments for the differences in their mechanisms. Taken together, these results demonstrated that *in vivo* treatment with ligustchuane B could attenuate the virulence of the *S. aureus* USA300 LAC strain.

## Discussion

Despite a rapid increase in multidrug-resistant bacterial strains, shortage of new antibiotics remains a major issue to be solved as only two novel classes were introduced in the past 20 years. As a result, new discovered antibiotics are insufficient to address the growing crisis of bacterial resistance (Jensen et al., 2008). For these reasons, a new promising strategy, i.e., anti-virulence therapy, was deemed necessary and desperately needed. Unlike antibiotics, anti-virulence therapy mainly targets the virulence factors secreted by bacteria for its drug action. This avoids posing selective pressure to the bacteria, thereby not affecting its growth which could lead to drug resistance.

In recent years, Hla has been the primary research focus among many virulence factors as it was found to be strongly associated with erythrocytes hemolysis, skin necrosis, and even some life-threatening diseases (Berube and Bubeck Wardenburg, 2013). Although the specific active constituents and the corresponding mechanism of actions of traditional Chinese medicine are poorly studied, it is undeniably that traditional Chinese medicine plays a pivotal role in clinical treatment of infectious disease in China. Thus, in this study traditional Chinese medicine extract library was screened and we have revealed for the first time that chuanxiong exhibited anti-infective activity against *S. aureus* by suppressing the expression of Hla. With further bioactivity-guided fractionation, a pair of unusual epimeric lignan trimers, ligustchuanes A and B, were characterized. Notably, the two lignan trimers were composed of C8-C9″-type neolignan, ferulic acid and ethoxyl groups, which have not been identified in any natural products.

In the biological evaluation, given the outstanding antihemolytic activity of ligustchuane B against MRSA, molecular docking studies were also conducted to speculate its possible interaction with *α*-hemolysin heptamer. The most likely binding interactions for (7*R*,8*R*) and (7*S*,8*S*)-enantiomers are shown in Supplementary Figure S15. The results revealed that both enantiomers of ligustchuane B could bind well to the *α*-hemolysin heptamer, suggesting that ligustchuane B may attenuate the virulence of *S. aureus* partly by direct interaction with heptamer protein. Moreover, a comparison of the binding affinity of these enantiomers indicated that the (7*S*,8*S*)-enantiomer (−8.5 kJ/mol) had a stronger binding affinity than the (7*R*,8*R*)-enantiomer (−7.6 kJ/mol). Even though the antihemolytic activities of both (7*S*,8*S*) and (7*R*,8*R*)-enantiomers were not determined in this study due to their small quantity obtained after enantioseparation, we speculated that the (7*S*,8*S*)-enantiomer may show a stronger inhibitory effect because of its higher binding affinity.

In conclusion, we have discovered a natural potential small-molecule *α*-hemolysin inhibitor isolated from the rhizomes of *Ligusticum chuanxiong* Hort, which could effectively attenuate the virulence of the *S. aureus* both *in vitro* and *in vivo*. Moreover, this study not only suggested innovative lead compounds for anti-virulence therapy against *S. aureus* but also further explored the chemical components in chuanxiong and expanded its traditional medicinal use.

## Data Availability

The datasets presented in this study can be found in online repositories. The names of the repository/repositories and accession number(s) can be found in the article/Supplementary Material.
