# Supplementary material for: Two Novel Phenylpropanoid Trimers From Ligusticum chuanxiong Hort With Inhibitory Activities on Alpha-Hemolysin Secreted by Staphylococcus aureus
Source: Front Chem. 2022 Mar 30;10:877469. doi: 10.3389/fchem.2022.877469 (PMC9006876; doi:10.3389/fchem.2022.877469)
Supplement: Supplementary file 1 [file DataSheet1.PDF]

Supplementary Material

Two Novel Phenylpropanoid Trimers from *Ligusticum chuanxiong* Hort with Inhibitory Activities on Alpha-hemolysin Secreted by *Staphylococcus aureus*

Shi-Jie Wan [1,3,4,†], Han-Gui Ren [1,3,†], Jia-Ming Jiang [1,3], Gang Xu [4], Yu Xu [1,3], Si-Min Chen [1,3], Gan Chen [1,3], Dan Zheng [5], Man Yuan [1,3], Hong Zhang [1,3,*] and Hong-Xi Xu [1,2,3,*]

[1] School of Pharmacy, Shanghai University of Traditional Chinese Medicine, Shanghai, 201203, P.R. China

[2] Shuguang Hospital, Shanghai University of Traditional Chinese Medicine, Shanghai, 201203, P.R. China

[3] Engineering Research Center of Shanghai Colleges for TCM New Drug Discovery, Shanghai, 201203, P.R. China

[4] State Key Laboratory of Phytochemistry and Plant Resources in West China and Yunnan Key Laboratory of Natural Medicinal Chemistry, Kunming Institute of Botany, Chinese Academy of Sciences, Kunming 650201, China

[5] Center for Translational Medicine and Shanghai Key Laboratory of Diabetes Mellitus, Shanghai JiaoTong University Affiliated Sixth People’s Hospital, Shanghai 200233, China

[*] Corresponding authors. E-mail addresses: xuhongxi88@gmail.com (H.X. Xu), zhnjau19851010@163.com (H. Zhang)

[†] These authors have contributed equally to this work and share first authorship.

List of Supplementary Information

Part 1 Experimental Section

1. Analysis of compounds **1** and **2** in a new plant extract by UPLC-ESI-QTOF-MS

2. Computational details

Figure S1. The stable conformers of (7*R*,8*S*)-**1** calculated with DFT at the B3LYP/6-31G (d, g) level.

Table S1. Optimized Z-matrixes of (-) ligustchuane A (-**1**) in the gas phase (Å) at B3LYP/6-31G(d, g) level.

Figure S2. The stable conformers of (7*R*,8*R*)-**2** calculated with DFT at the B3LYP/6-31G (d, g) level.

Table S2. Optimized Z-matrixes of (+) ligustchuane B (+**2**) in the gas phase (Å) at B3LYP/6-31G(d, g) level.

Part 2 Supplementary Figures

Figure S3. LCA inhibits the expression of Hla in both Newman and USA300 LAC strain.

Figure S4. Effect of LCA (100 mg/kg/d) on the survival of the *S. aureus* Newman strain in the spleens, livers, kidneys and hearts of mice (n = 5) intraperitoneally challenged with 1 × 10^8 CFU of bacteria.

Figure S5. LCA, LCAII and senkyunolide A have no toxic effect on the growth of *S. aureus*.

Figure S6. LCAII shows substantial inhibition against Hla in both Newman and USA300 LAC strain.

Figure S7. LCAI and LCAIII show weak inhibitory effect on Hla expression.

Figure S8. Effect of LCAII (100 mg/kg/d) on the survival of the *S. aureus* USA300 LAC strain in the spleens, livers and kidneys of mice (n = 7) intraperitoneally challenged with 7.4×10^8 CFU of bacteria.

Figure S9. Identification of two main constituents in LCAII by UPLC-PDA-QTOF-MS.

Figure S10. Senkyunolide A reduces hemolytic activity of *S. aureus* by inhibiting Hla production.

Figure S11. *Z*-ligustilide has no effect on Hla expression.

Figure S12. LCAIIB shows outstanding inhibition against Hla in Newman strain.

Figure S13. UPLC-ESI-QTOF-MS analysis of compounds **1** and **2** in a new plant extract of chuanxiong.

Figure S14. Ligustchuanes A (**1**) and B (**2**) have no toxic effect on the growth of *S. aureus*.

Figure S15. Compounds (±) **2** may directly interact with α-hemolysin heptamer of *S. aureus*.

Part 3 HRESIMS, IR, ECD, and NMR spectra of compounds 1 and 2

Ligustchuane A (**1**)

Figure S16. HRESIMS spectrum of **1**

Figure S17. IR (KBr, disc) spectrum of **1**

Figure S18. Experimental ECD spectrum of **1**

Figure S19. Experimental ECD spectrum of **(+) 1**

Figure S20. Experimental ECD spectrum of **(-) 1**

Figure S21. ^{1}H NMR spectrum (DMSO-d_6, 600 MHZ) of **1**

Figure S22. ^{13}C and DEPT-135 spectra (DMSO-d_6, 150 MHZ) of **1**

Figure S23. HSQC NMR spectrum (DMSO-d_6, 600 MHZ, 150 MHZ) of **1**

Figure S24. HMBC NMR spectrum (DMSO-d_6, 600 MHZ, 150 MHZ) of **1**

Figure S25. ^{1}H-^{1}H COSY NMR spectrum (DMSO-d_6, 600 MHZ) of **1**

Figure S26. NOESY NMR spectrum (DMSO-d_6, 600 MHZ) of **1**

Figure S27. Enantioseparation via chiral-phase UPC^2 on a Daicel Chiralpak IG column of **1**

Ligustchuane B (**2**)

Figure S28. HRESIMS spectrum of **2**

Figure S29. IR (KBr, disc) spectrum of **2**

Figure S30. Experimental ECD spectrum of **2**

Figure S31. Experimental ECD spectrum of **(+) 2**

Figure S32. Experimental ECD spectrum of **(-) 2**

Figure S33. ^{1}H NMR spectrum (DMSO-d_6, 400 MHZ) of **2**

Figure S34. ^{13}C and DEPT-135 spectra (DMSO-d_6, 100 MHZ) of **2**

Figure S35. HSQC NMR spectrum (DMSO-d_6, 400 MHZ, 100 MHZ) of **2**

Figure S36. HMBC NMR spectrum (DMSO-d_6, 400 MHZ, 100 MHZ) of **2**

Figure S37. ^{1}H-^{1}H COSY NMR spectrum (DMSO-d_6, 400 MHZ) of **2**

Figure S38. NOESY NMR spectrum (DMSO-d_6, 400 MHZ) of **2**

Figure S39. Enantioseparation via chiral-phase UPC^2 on a Daicel Chiralpak IG column of **2**

Supplementary information Available

Part 1. Experimental section

1. Analysis of compounds **1** and **2** in a new plant extract by UPLC-ESI-QTOF-MS

1.1 Sample preparation

The Air-dried and fragmented rhizomes of *Ligusticum chuanxiong* Hort (20 g, with the same origin that the plant material used for the initial extraction) were extracted by heat reflux with 80% MeOH (3 × 0.2 L). The obtained solutions were combined and concentrated in a rotary evaporator at 45 °C. The residue was suspended in H_2O and extracted with PE to obtain PE-soluble fraction named LCD. After filtration through 0.22 μm microporous membrane, 5 μL of the sample was injected for UPLC-ESI-QTOF-MS analysis.

1.2 UPLC-PDA-QTOF-MS analysis

A Waters ACQUITY UPLC TM system (Waters corporation, Milford, MA, USA) was used to perform UPLC analysis, with a binary solvent delivery system and an autosampler. Samples were eluted on a Waters ACQUITY BEH C_{18} column (100 mm × 2.1 mm, 1.7 μm) maintained at 40 °C. 0.1% formic acid in water (A) and CH_3CN (B) was applied as mobile phase. The elution gradient and flow rate were set as follows: 0-5 min: 5% B to 15% B, 5-15 min: 15% B to 22% B, 15-20 min: 22% B to 28% B, 20-30 min: 28% B to 65% B, 30-35 min: 65% B to 90% B with the flow rate of 0.4 mL/min. All fractions or compounds were analyzed using the data-dependent acquisition (DDA) mode. The top five ions were selected for MS/MS from a single MS survey scan. The MS parameters were set as follows: ESI positive mode and negative mode; mass range: 50-1200 Da; desolvation gas, 800 L/h; cone gas: 50 L/h; desolvation gas temperature: 400 °C; source temperature: 120 °C; capillary voltage: 2500 V; time segments: 1-5 min (LC flow to waste); 5-25 min (LC flow to MS); data format: centroid. The SYNAPT G2-Si HDMS system was calibrated using sodium formate clusters and operated in resolution mode. The molecular masses of the precursor ion and the product ions were accurately determined using

leucine-enkephalin as a reference compound (*m/z* 556.2771) in the LockSpray mode at a concentration of 50 pg/μL at an infusion flow rate of 5 μL/min.

MS/MS collision energy: 20 V to 50 V, scan time: 0.1 s, data format: centroid. The capillary voltage was set to 3000 V. Spectra were acquired in centroid and positive mode. Argon was employed as the collision gas. Other parameters were set as described in MS parameters.

2. Computational details

All calculations were performed using Gaussian 09.[1] Conformational search was initially carried out using Accelrys Discovery Studio 2.5 to generate conformations by Best with 10 kcal/mol upper energy limit, then was minimized by Smart Minimizer using the CHARMm molecular mechanics force field. The conformers were further optimized at the B3LYP/6-31G (d, g) level in the gas phase. Room-temperature equilibrium populations were calculated according to the Boltzmann distribution law. The theoretical calculation of ECD was performed using TDDFT at the B3LYP/6-31G (d, p) level in the gas phase. By weighing the Boltzmann distribution rate of each geometric conformation, the ECD spectra of **1** and **2** were obtained. SpecDis 1.61[2] was later used to sum up single CD spectra after a Boltzmann statistical weighting, for the gauss curve generation and for the comparison with experimental data.

References:

(1) Gaussian 09, Revision D.01, M. J. Frisch, G. W. Trucks, H. B. Schlegel, G. E. Scuseria, M. A. Robb, J. R. Cheeseman, G. Scalmani, V. Barone, B. Mennucci, G. A. Petersson, H. Nakatsuji, M. Caricato, X. Li, H. P. Hratchian, A. F. Izmaylov, J. Bloino, G. Zheng, J. L. Sonnenberg, M. Hada, M. Ehara, K. Toyota, R. Fukuda, J. Hasegawa, M. Ishida, T. Nakajima, Y. Honda, O. Kitao, H. Nakai, T. Vreven, J. A. Montgomery, Jr., J. E. Peralta, F. Ogliaro, M. Bearpark, J. J. Heyd, E. Brothers, K. N. Kudin, V. N. Staroverov, T. Keith, R. Kobayashi, J. Normand, K. Raghavachari, A. Rendell, J. C. Burant, S. S. Iyengar, J. Tomasi, M. Cossi, N. Rega, J. M. Millam, M. Klene, J. E. Knox, J. B. Cross, V. Bakken, C. Adamo, J. Jaramillo, R. Gomperts, R. E. Stratmann, O. Yazyev, A. J. Austin, R. Cammi, C. Pomelli, J. W. Ochterski, R. L. Martin, K. Morokuma, V. G. Zakrzewski, G. A. Voth, P. Salvador, J. J. Dannenberg, S. Dapprich, A. D. Daniels, O. Farkas, J. B. Foresman, J. V. Ortiz, J. Cioslowski, and D. J. Fox, Gaussian, Inc., Wallingford CT, 2013.

(2) T. Bruhn, A. Schaumlöffel, Y. Hemberger, G. Bringmann, SpecDis version 1.64,

University of Wuerzburg, Germany, 2015.

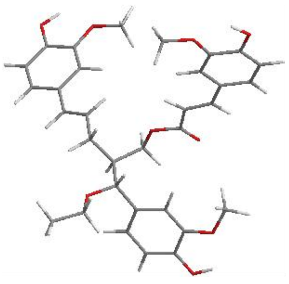

(−) 1a (39.23)

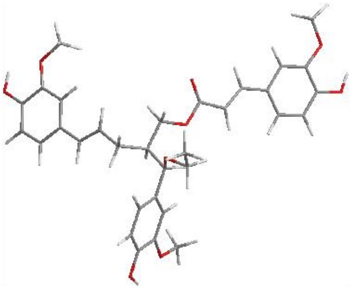

(−) 1b (39.23)

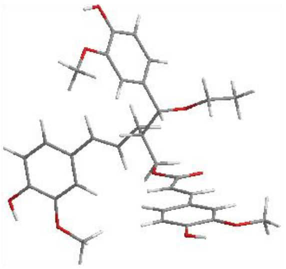

(−) 1c (12.34)

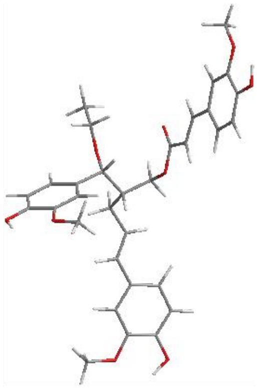

(−) 1d (4.42)

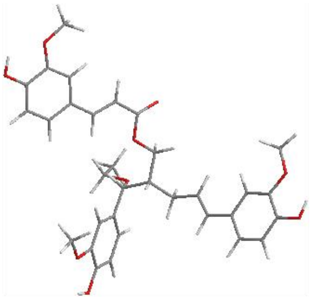

(−) 1e (2.82)

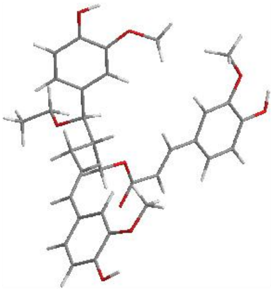

(−) 1f (1.97)

Figure S1. The stable conformers of (7*R*,8*S*)-**1** calculated with DFT at the B3LYP/6-31G (d, g) level. Relative populations are in parentheses. Equilibrium Populations calculated by the relative free Gibbs energies at B3LYP/6-31G (d) level in the gas phase, assuming Boltzman statistics at T = 298.15 K and 1 atm.

Table S1. Optimized Z-matrixes of (-) ligustchuane A (-**1**) in the gas phase (Å) at B3LYP/6-31G(d, g) level

(-) 1a				(-) 1b			
C	-3.43940300	-5.12004400	-1.03586800	C	-0.61651500	5.89557800	-1.41567500
C	-3.73282600	-4.84457500	0.29368400	C	0.59843000	5.94421300	-0.74038200
C	-2.93627100	-3.95255900	1.01924100	C	0.96087500	4.91004300	0.12617700
C	-1.83557100	-3.33198900	0.42770100	C	0.10810700	3.82461400	0.34090000
C	-1.53274400	-3.61886600	-0.91480200	C	-1.12777000	3.78729600	-0.32484200
C	-2.32797900	-4.49727500	-1.64148700	C	-1.48133700	4.80214400	-1.20812600
C	-0.96589600	-2.33734400	1.18775500	C	0.53025200	2.67388500	1.24522100
O	-1.03252400	-2.51628400	2.59887300	O	1.25020500	3.13780800	2.38458100

C	-1.33029500	-0.85753700	0.89806500	C	1.45319200	1.65150700	0.52887300
C	-2.72804300	-0.47836600	1.45283100	C	1.97722500	0.57904300	1.52211800
C	-0.25345700	0.07724800	1.44428100	C	0.77501800	1.03530500	-0.69469200
C	-3.20055200	0.89496600	1.05774700	C	2.86898300	-0.45765400	0.89715000
C	-4.17279200	1.12322500	0.16054400	C	4.19271000	-0.53685900	1.10683500
C	-4.69819000	2.41503900	-0.29966200	C	5.14388100	-1.50099300	0.53878400
C	-5.74803200	2.43224500	-1.22816700	C	6.46568000	-1.51224600	1.00495300
C	-6.28656700	3.63001300	-1.70181100	C	7.41011800	-2.41083300	0.50553500
C	-5.78104300	4.84282300	-1.25227100	C	7.04868400	-3.31945600	-0.48065300
C	-4.72221600	4.84451300	-0.31711900	C	5.72247900	-3.31804100	-0.96698200
C	-4.18906900	3.65359300	0.15074300	C	4.78676200	-2.42456700	-0.46895600
O	-6.29479300	6.01937700	-1.70512800	O	7.95959300	-4.19874200	-0.98091100
C	-0.26205200	-3.60826600	3.09222800	C	0.44965000	3.80110500	3.35616400
C	-0.40727200	-3.64687200	4.60364200	C	1.34942100	4.21748500	4.50722800
O	0.98431900	-0.20893200	0.75488100	O	-0.27663400	0.14222800	-0.24908600
C	2.02661000	0.61179900	1.04482800	C	-1.37162000	0.00413000	-1.03940400
C	3.22676900	0.22015200	0.28415700	C	-2.31032000	-0.96435000	-0.44838800
O	1.94683700	1.53986000	1.83030700	O	-1.53615000	0.61226800	-2.08480900
C	4.37346200	0.91179500	0.43209300	C	-3.45599000	-1.27870700	-1.08615900
C	5.65529800	0.68451400	-0.22511600	C	-4.49638700	-2.20657500	-0.66469200
C	6.73145300	1.54199400	0.09601600	C	-4.43272400	-2.92469500	0.55295000
C	7.97339200	1.37240100	-0.49782800	C	-5.45067900	-3.79423100	0.90356200
C	8.17624900	0.33669000	-1.43420000	C	-6.56683100	-3.97611200	0.05125300
C	7.12008200	-0.51328200	-1.75738400	C	-6.63688600	-3.27364000	-1.14649700
C	5.87559800	-0.34115900	-1.15996200	C	-5.61017700	-2.39867000	-1.49689800
O	9.39055500	0.16965400	-2.01554400	O	-7.55731600	-4.83042200	0.40555700
O	-4.21096200	-5.98782400	-1.74890500	O	-0.97103500	6.89321600	-2.27284100
O	-4.31540300	6.10925900	0.04161400	O	5.50171300	-4.25936100	-1.94652400
C	-3.25337800	6.23348800	0.97560400	C	4.20938700	-4.33353300	-2.52858100
O	9.09406200	2.13384600	-0.27204800	O	-5.51631600	-4.54945500	2.04765100
C	9.00361100	3.20399700	0.65907500	C	-4.44683600	-4.45576500	2.97965000
O	-2.14216800	-4.86070300	-2.95563200	O	-2.64231000	4.86550400	-1.93920700
C	-1.05981900	-4.27892600	-3.66610600	C	-3.49275700	3.72213500	-1.94063000
H	-4.58760200	-5.33467300	0.74819000	H	1.25218900	6.79336000	-0.91080200
H	-3.16565100	-3.74160100	2.05801300	H	1.90915100	4.94987300	0.65211200
H	-0.67073200	-3.14961100	-1.37813500	H	-1.79969800	2.95220600	-0.16158000
H	0.07500900	-2.47530300	0.85711400	H	-0.37242200	2.13548300	1.58041800
H	-1.35490900	-0.74062700	-0.19264100	H	2.31781600	2.20855500	0.14319600
H	-2.69894500	-0.56913400	2.54592400	H	1.10710800	0.08679100	1.97696500
H	-3.44715600	-1.22195500	1.09522800	H	2.50765500	1.09709300	2.32358100
H	-0.10960400	-0.07530400	2.51805100	H	1.49243700	0.44068300	-1.26858400
H	-0.50665000	1.12694400	1.27883500	H	0.34825200	1.78812600	-1.35691000
H	-2.71188000	1.73800800	1.54474700	H	2.38162400	-1.18091900	0.24426700

H	-4.65108100	0.25572200	-0.29605400	H	4.63944300	0.18734300	1.78928000
H	-6.15309300	1.49035900	-1.58712000	H	6.76098000	-0.80662100	1.77630500
H	-7.09950200	3.63890900	-2.42015100	H	8.43204800	-2.41688900	0.86961200
H	-3.37399000	3.67215300	0.86389300	H	3.77713700	-2.42644300	-0.86154900
H	-5.80039400	6.72932500	-1.26642600	H	7.50241200	-4.72412600	-1.65603500
H	-0.60672200	-4.55152500	2.64446200	H	-0.04324100	4.67781800	2.91243600
H	0.79384700	-3.47468700	2.80663800	H	-0.34417700	3.12216600	3.70980200
H	-1.45317400	-3.79653500	4.88730000	H	0.76566500	4.72064000	5.28448200
H	0.18483900	-4.46659500	5.02242400	H	1.83888400	3.34521800	4.95038100
H	-0.06345900	-2.70812000	5.04766600	H	2.12561100	4.90505400	4.15897300
H	3.12881500	-0.63258100	-0.37914800	H	-2.02047500	-1.40131600	0.50125300
H	4.33572900	1.74583900	1.13166700	H	-3.62570000	-0.77860100	-2.03850600
H	6.57126700	2.33687900	0.81582400	H	-3.58330700	-2.79010000	1.21123600
H	7.29536800	-1.30352400	-2.47952200	H	-7.49786100	-3.42407100	-1.78853200
H	5.06755100	-1.01394400	-1.42632100	H	-5.67090500	-1.85373300	-2.43420300
H	9.97600400	0.85531200	-1.65635900	H	-7.31394800	-5.21222500	1.26413100
H	-3.83338700	-6.03335000	-2.64094300	H	-1.83083000	6.64605800	-2.64813300
H	-3.51289600	5.77565200	1.93804600	H	3.93749300	-3.39073000	-3.01900800
H	-3.09422100	7.30350900	1.11558200	H	3.44761300	-4.58237200	-1.77941800
H	-2.33193100	5.77542300	0.59581700	H	4.25579200	-5.12848700	-3.27410400
H	8.74360800	2.83928000	1.66007700	H	-3.49791500	-4.76783300	2.52732700
H	8.26276800	3.94713200	0.34037800	H	-4.69753200	-5.13078600	3.79844400
H	9.99088600	3.66628400	0.68723600	H	-4.34605600	-3.43482200	3.36699900
H	-1.15267500	-3.18683800	-3.71114800	H	-2.94576800	2.81880000	-2.23307600
H	-1.10333600	-4.68759200	-4.67653700	H	-4.27935300	3.93288300	-2.66668100
H	-0.09644300	-4.53985600	-3.21068000	H	-3.94826700	3.56823300	-0.95413500
**(-) 1c**				**(-) 1d**			
C	1.83850300	5.33173500	-0.92454800	C	1.98485900	4.78145700	-1.24497300
C	2.08589900	5.15063400	0.43009300	C	2.16054300	4.76785800	0.13266300
C	1.58783900	4.02278500	1.09093200	C	1.56751700	3.76504900	0.90721300
C	0.83415400	3.06809500	0.40826100	C	0.78969200	2.77011000	0.31532800
C	0.57568600	3.25590600	-0.96106600	C	0.60296800	2.78975900	-1.07821000
C	1.07596900	4.37160900	-1.62206800	C	1.19762700	3.78013000	-1.85107000
C	0.30068600	1.82035900	1.10401400	C	0.15597600	1.65143500	1.13543800
O	0.22923000	1.97495500	2.51482200	O	0.04164000	1.98428200	2.51170400
C	1.14625600	0.55083400	0.82699300	C	0.93531800	0.31340200	1.05187200
C	2.55125300	0.63826300	1.47749800	C	2.31322300	0.39947400	1.75808800
C	0.39582200	-0.69614200	1.30341900	C	0.09200700	-0.82679800	1.63020300
C	3.49328600	-0.46625400	1.08053700	C	3.21661600	-0.77519800	1.49708800
C	4.55424100	-0.29125900	0.27660700	C	4.32472700	-0.71273000	0.74207700
C	5.53099800	-1.29159400	-0.17257000	C	5.25801500	-1.80088900	0.41769800
C	6.56894200	-0.89406000	-1.02663000	C	5.01165700	-3.14982800	0.71689900
C	7.52560100	-1.80028300	-1.48746100	C	5.93405200	-4.14239100	0.39183200

C	7.46270300	-3.13207300	-1.09883200	C	7.12632200	-3.81191300	-0.24590500
C	6.42435700	-3.55036400	-0.23728400	C	7.38724000	-2.46410900	-0.56066600
C	5.47541800	-2.64819000	0.21789000	C	6.46592400	-1.47660700	-0.23565300
O	8.38839100	-4.02779800	-1.53926400	O	8.02789300	-4.78064800	-0.56755400
C	-0.95306200	2.62586700	2.97857800	C	-1.12052600	2.74419400	2.84241100
C	-0.87981200	2.71416100	4.49263200	C	-1.10304900	3.00354400	4.33847600
O	-0.76804500	-0.92870700	0.47519200	O	-1.03748000	-1.10261200	0.76862300
C	-1.99251400	-0.89845700	1.06582700	C	-2.28800600	-0.90735800	1.26503800
C	-3.04175000	-1.21121900	0.07947200	C	-3.29800400	-1.28588600	0.26103900
O	-2.17899400	-0.64436700	2.24378200	O	-2.52267600	-0.47470100	2.38070300
C	-4.33491300	-1.23680000	0.45796400	C	-4.60988000	-1.15943500	0.54220000
C	-5.50800600	-1.52984200	-0.35658300	C	-5.74882600	-1.48003000	-0.30987600
C	-6.77512700	-1.51211600	0.26859900	C	-7.04745800	-1.23989800	0.19294900
C	-7.92581200	-1.78574900	-0.45569600	C	-8.16694800	-1.52304300	-0.57534400
C	-7.84273100	-2.08449700	-1.83223000	C	-8.01994800	-2.05796100	-1.87270700
C	-6.59695000	-2.10360100	-2.45708100	C	-6.74280000	-2.29956400	-2.37578800
C	-5.44425600	-1.82998400	-1.72796400	C	-5.62160200	-2.01398100	-1.60357300
O	-8.96738100	-2.35164900	-2.54186800	O	-9.11428900	-2.33570500	-2.62454200
O	2.32012500	6.42811900	-1.57556400	O	2.56075500	5.75435700	-2.00702700
O	6.47931000	-4.89096800	0.06982800	O	8.59517800	-2.27035400	-1.19205000
C	5.49097200	-5.42236200	0.93875000	C	8.96242300	-0.94591100	-1.54576100
O	-9.20950700	-1.80259600	0.03239600	O	-9.47649400	-1.33546000	-0.20624600
C	-9.40723300	-1.52517400	1.41247400	C	-9.74261100	-0.80788900	1.08646700
O	0.89693900	4.67364700	-2.95300100	O	1.09692600	3.91192500	-3.21755600
C	0.15230100	3.76763100	-3.75220600	C	0.33328200	2.95021500	-3.92906600
H	2.66931600	5.89975300	0.95536300	H	2.76338000	5.54787900	0.58620900
H	1.77754700	3.88385500	2.14937900	H	1.70097100	3.75669700	1.98319100
H	-0.02128200	2.52468000	-1.49681600	H	-0.01215600	2.02683300	-1.54484600
H	-0.70766600	1.62369000	0.70756500	H	-0.84678200	1.46301900	0.72148400
H	1.27737800	0.47410800	-0.25960700	H	1.10896800	0.10140100	-0.01045300
H	2.42296400	0.65478900	2.56711000	H	2.13971900	0.51500400	2.83537200
H	2.99190500	1.60106000	1.20030600	H	2.80991900	1.31513800	1.42174700
H	0.08289100	-0.58685000	2.34267900	H	-0.26751300	-0.57454700	2.62884000
H	1.01639800	-1.58897700	1.19249100	H	0.66578500	-1.75623100	1.66460600
H	3.28520200	-1.45632700	1.48540500	H	2.94194700	-1.72138500	1.96173200
H	4.73733000	0.71502500	-0.10223300	H	4.58920200	0.25672900	0.31703200
H	6.63012700	0.14500300	-1.33747600	H	4.08371800	-3.43679500	1.20018600
H	8.32732800	-1.48798900	-2.14826000	H	5.74207400	-5.18544800	0.62093800
H	4.68587600	-2.98448900	0.87871500	H	6.66925700	-0.43885900	-0.47694600
H	8.15853700	-4.88525500	-1.14845200	H	8.77468800	-4.33740300	-0.99949100
H	-1.03132300	3.62916400	2.53383600	H	-1.12463800	3.69185300	2.28331700
H	-1.83271500	2.04342500	2.66943100	H	-2.01768900	2.17482100	2.56129800
H	-0.00613300	3.29289900	4.80741600	H	-0.21232300	3.57051700	4.62577300

H	-1.77710500	3.20049700	4.88830100	H	-1.98757700	3.57617000	4.63502400
H	-0.80748600	1.71399200	4.92905400	H	-1.10288800	2.05796800	4.88787900
H	-2.71354200	-1.42057700	-0.93290300	H	-2.92625200	-1.66304400	-0.68553200
H	-4.52523900	-1.01219200	1.50659600	H	-4.84718000	-0.76785400	1.53048700
H	-6.83397000	-1.28220000	1.32660300	H	-7.15574700	-0.82900400	1.19049800
H	-6.55358700	-2.33628500	-3.51568200	H	-6.65051600	-2.71316800	-3.37431800
H	-4.48507000	-1.85147600	-2.23397600	H	-4.63695200	-2.21209100	-2.01299800
H	-9.71530800	-2.28867600	-1.92658800	H	-9.89249500	-2.10022800	-2.09453000
H	2.03248200	6.35622300	-2.49882500	H	2.30799100	5.57772200	-2.92639000
H	5.52339100	-4.93914300	1.92306700	H	8.24670500	-0.50743800	-2.25217000
H	5.72023600	-6.48311100	1.04905000	H	9.94129700	-1.01765200	-2.02187000
H	4.48507000	-5.31040100	0.51554700	H	9.03527500	-0.30156900	-0.66095300
H	-8.88901300	-2.25752400	2.04313100	H	-9.30898900	0.19243600	1.20501600
H	-10.48173200	-1.59400900	1.58527600	H	-9.35460100	-1.46665700	1.87264200
H	-9.06112000	-0.51670700	1.66896200	H	-10.82797000	-0.74390500	1.17074800
H	0.62101500	2.77601600	-3.77415000	H	0.74134400	1.94033200	-3.79826600
H	0.14348600	4.18557000	-4.75979200	H	0.39293600	3.23093100	-4.98150000
H	-0.87904900	3.67021300	-3.39104400	H	-0.71648200	2.95790900	-3.61053000
**(-) 1e**				**(-) 1f**			
C	-1.28513600	5.39175500	1.13211300	C	-0.18395900	5.35641100	-1.17926700
C	-1.79510400	5.29942100	-0.15671100	C	0.50724600	5.39485700	0.02511400
C	-1.43563200	4.22725900	-0.97993100	C	0.52443600	4.27423100	0.86164200
C	-0.55898700	3.24020200	-0.52827200	C	-0.15309900	3.10625500	0.50986400
C	-0.03604200	3.33880400	0.77297600	C	-0.86052400	3.07099300	-0.70463300
C	-0.39898900	4.39771800	1.59724000	C	-0.87121900	4.17988300	-1.54308700
C	-0.17197900	2.04830500	-1.39632200	C	-0.12395800	1.86340700	1.39259300
O	-0.32286200	2.31021400	-2.78725800	O	0.18078000	2.16486500	2.74925600
C	-0.99153600	0.76907900	-1.08177700	C	0.90358200	0.80226600	0.92030000
C	-2.48791200	0.92531900	-1.45854900	C	2.36622600	1.28675100	1.07157300
C	-0.38195100	-0.44702200	-1.77518200	C	0.70602000	-0.51540200	1.66467600
C	-3.36305300	-0.21341000	-1.00865600	C	3.36965400	0.40481900	0.38200300
C	-4.25856200	-0.12246900	-0.01268000	C	4.39530400	-0.20926700	0.99279100
C	-5.14871600	-1.16991800	0.50432300	C	5.43208900	-1.05929200	0.39320800
C	-6.07711900	-0.84455600	1.50265800	C	6.38187000	-1.66550800	1.22679700
C	-6.95021600	-1.79849300	2.02875500	C	7.38765400	-2.48633400	0.71389100
C	-6.90965400	-3.10643200	1.56369700	C	7.46419400	-2.71703700	-0.65346700
C	-5.97784300	-3.45306800	0.56005600	C	6.51713700	-2.11162200	-1.50884600
C	-5.11164400	-2.50385100	0.04028900	C	5.52004200	-1.29600100	-0.99677300
O	-7.75304700	-4.04858500	2.06789200	O	8.44017800	-3.51384400	-1.16863900
C	0.71423400	3.10533900	-3.35495400	C	-0.91226900	2.68680700	3.49982300
C	0.43969800	3.25257200	-4.84153900	C	-0.44199700	2.91734600	4.92549700
O	0.94757100	-0.65499400	-1.24608500	O	-0.59705000	-1.04166500	1.32142800
C	1.56762000	-1.79450300	-1.64769500	C	-0.95117000	-2.18647000	1.96273100

C	2.91941200	-1.95924400	-1.08735100	C	-2.28578900	-2.68605900	1.58784700
O	1.05104800	-2.60369300	-2.39811900	O	-0.23159300	-2.74529200	2.77018800
C	3.52018600	-1.08218200	-0.25876800	C	-3.11172000	-2.08680000	0.70813900
C	4.85117800	-1.18181300	0.33117500	C	-4.44105600	-2.53012400	0.29426000
C	5.70295000	-2.28982600	0.11318200	C	-5.17396500	-1.71226800	-0.59446700
C	6.95768300	-2.33135200	0.69607600	C	-6.44223200	-2.08344100	-1.01712000
C	7.40592000	-1.26831500	1.51640200	C	-7.01600300	-3.28992900	-0.56486400
C	6.57443300	-0.17638200	1.73659500	C	-6.29869600	-4.10439800	0.30922100
C	5.31076500	-0.13835500	1.14883100	C	-5.02797200	-3.72895200	0.73313700
O	8.63722500	-1.32085600	2.08196600	O	-8.25534100	-3.65555100	-0.97888300
O	-1.63053000	6.43347600	1.93939900	O	-0.20433400	6.44447700	-1.99916300
O	-6.03918200	-4.77834600	0.19509200	O	6.70866800	-2.41904500	-2.83701000
C	-5.14843000	-5.24039800	-0.80948800	C	5.81373400	-1.86661200	-3.78935700
O	7.87836400	-3.34149700	0.56974200	O	-7.25363400	-1.37789300	-1.87363100
C	7.53611600	-4.47059800	-0.22385200	C	-6.78021700	-0.13905200	-2.38013100
O	0.03751500	4.60910100	2.88493200	O	-1.52034200	4.27423900	-2.75406700
C	0.90827200	3.65006700	3.46472200	C	-2.20709700	3.13188300	-3.23690300
H	-2.47286400	6.07293200	-0.50268300	H	1.02854800	6.30743600	0.29483700
H	-1.83397300	4.15909400	-1.98636100	H	1.06335100	4.30713800	1.80202500
H	0.65465400	2.58058000	1.12840100	H	-1.39984300	2.17030000	-0.98082600
H	0.88232100	1.80683800	-1.18971200	H	-1.11546300	1.38660400	1.34376200
H	-0.92872700	0.60628500	0.00155900	H	0.70469700	0.62300600	-0.14525600
H	-2.55638300	1.04841500	-2.54691200	H	2.60867200	1.37987700	2.13554800
H	-2.85401700	1.85615700	-1.01460600	H	2.42702400	2.29801300	0.64856000
H	-0.31599200	-0.28796600	-2.85557200	H	0.75970300	-0.36379500	2.74584800
H	-0.96935700	-1.35023200	-1.59733600	H	1.46952400	-1.24538800	1.38410700
H	-3.24975700	-1.15629000	-1.54234800	H	3.22917900	0.28716800	-0.69348100
H	-4.36235500	0.84197800	0.48621900	H	4.49334500	-0.07401700	2.07040400
H	-6.12030700	0.17550700	1.87371000	H	6.33164200	-1.49529400	2.29840600
H	-7.66835900	-1.54237700	2.80052900	H	8.11986300	-2.95505000	1.36275700
H	-4.39685200	-2.78754900	-0.72259300	H	4.80596900	-0.83255400	-1.66668000
H	-7.55399700	-4.87904600	1.60783900	H	8.30733900	-3.53693800	-2.12914000
H	0.75035200	4.08997100	-2.86695800	H	-1.26924600	3.62554900	3.05170500
H	1.68817100	2.61821500	-3.18604800	H	-1.75004600	1.97149100	3.47810700
H	1.22542100	3.84837300	-5.31649800	H	-1.26191700	3.30419700	5.53865900
H	0.40639800	2.27248600	-5.32619100	H	-0.08729600	1.98293800	5.36976900
H	-0.51988300	3.75019400	-5.00985900	H	0.37805700	3.64102300	4.94988100
H	3.40039300	-2.87522200	-1.41448400	H	-2.55150700	-3.60335300	2.10245700
H	2.96069500	-0.18920800	0.01009100	H	-2.76121000	-1.16535200	0.24812600
H	5.36886400	-3.10912100	-0.51104800	H	-4.73078800	-0.78462900	-0.94004200
H	6.93109800	0.63042400	2.36758200	H	-6.75418900	-5.02999900	0.64447500
H	4.66750800	0.71887600	1.32480100	H	-4.48750800	-4.38162000	1.40986100
H	9.04440600	-2.15672700	1.80346700	H	-8.57588900	-2.95901100	-1.57353100

H	-1.16988700	6.30201000	2.78254600	H	-0.73570500	6.20570400	-2.77422500
H	-5.30689400	-4.71125800	-1.75713300	H	4.78218900	-2.18937100	-3.60149500
H	-5.36667100	-6.30035300	-0.94621000	H	6.13696000	-2.23745300	-4.76301600
H	-4.10251100	-5.12146700	-0.50124400	H	5.85415000	-0.77026200	-3.78646800
H	6.65374900	-4.98390900	0.17654700	H	-6.58911700	0.57568600	-1.57024400
H	8.39509900	-5.14109800	-0.18405400	H	-7.56984400	0.24814300	-3.02511900
H	7.34689600	-4.18207400	-1.26471700	H	-5.86398200	-0.27358500	-2.96837200
H	0.44076000	2.65852000	3.50417300	H	-1.52754400	2.27958600	-3.36188000
H	1.11043100	3.99540000	4.47946800	H	-2.61989500	3.41069200	-4.20750900
H	1.85297900	3.58116600	2.91098800	H	-3.02535900	2.84276300	-2.56496200

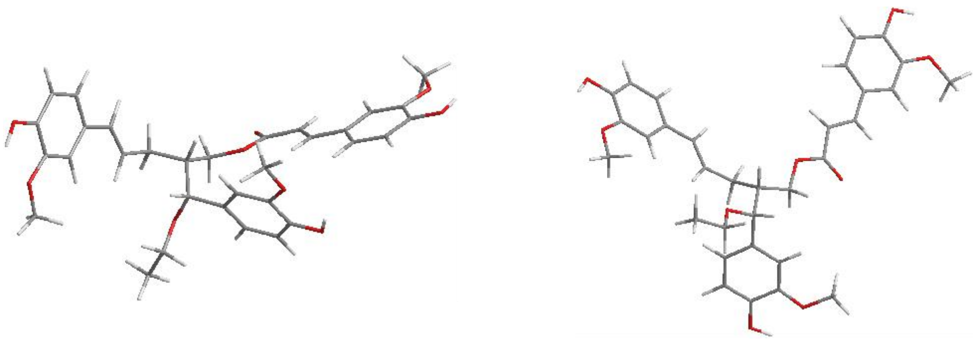

(+) 2a (50) **(+) 2b** (50)

Figure S2. The stable conformers of (7*R*,8*R*)-**2** calculated with DFT at the B3LYP/6-31G (d, g) level. Relative populations are in parentheses. Equilibrium Populations calculated by the relative free Gibbs energies at B3LYP/6-31G (d) level in the gas phase, assuming Boltzman statistics at T = 298.15 K and 1 atm.

Table S2. Optimized Z-matrixes of (+) ligustchuane B (+**2**) in the gas phase (Å) at B3LYP/6-31G(d, g) level

(+) 2a				(+) 2b			
C	-2.06499600	1.13891600	2.68960100	C	-2.81683600	-5.20059800	-1.58733600
C	-1.81304100	2.36039000	2.07746700	C	-3.58639700	-4.04702800	-1.68109400
C	-0.70156600	2.51061600	1.24359900	C	-3.37725100	-2.98809600	-0.79276100
C	0.17373000	1.44842300	1.01884400	C	-2.40300400	-3.06966600	0.20490400
C	-0.07469700	0.21545600	1.64471500	C	-1.64004500	-4.24607500	0.31121700
C	-1.18927500	0.05801600	2.46124500	C	-1.83380200	-5.29518200	-0.58088800
C	1.38338600	1.58613200	0.10203600	C	-2.14323600	-1.91086600	1.16326500
O	1.80683200	2.93877500	-0.04364300	O	-3.32935000	-1.17736800	1.45446700
C	1.16879600	1.02731100	-1.32739000	C	-1.07768700	-0.88644100	0.68182100
C	2.46503600	1.12736700	-2.18058500	C	-1.41289400	-0.13972400	-0.63526300

C	0.02308800	1.69906500	-2.09092000	C	0.28015800	-1.58358800	0.57432400
C	3.69304100	0.53441500	-1.54702800	C	-2.42351500	0.96708700	-0.50563800
C	4.22523000	-0.64643900	-1.90094300	C	-2.17266400	2.24380500	-0.83428100
C	5.41294400	-1.31201300	-1.35121600	C	-3.08482000	3.39251000	-0.74962400
C	5.76097600	-2.58387700	-1.82669800	C	-2.65725500	4.64291800	-1.21666700
C	6.87572400	-3.26782000	-1.33896300	C	-3.48347600	5.76717700	-1.16168000
C	7.67210000	-2.68643000	-0.36097200	C	-4.76277900	5.66201900	-0.63192400
C	7.33888600	-1.40312600	0.12706300	C	-5.20915200	4.41048900	-0.15388100
C	6.23000200	-0.72708900	-0.35792400	C	-4.38674200	3.29585600	-0.20994700
O	8.76342600	-3.33973100	0.12264000	O	-5.58028300	6.75010700	-0.56937100
C	2.48877200	3.47494000	1.08585500	C	-4.24204900	-1.85704800	2.31042000
C	2.88843500	4.90475100	0.76438400	C	-5.43810200	-0.95053800	2.54582400
O	-1.22531700	1.09820100	-1.69404000	O	1.29452300	-0.56654400	0.46593800
C	-2.28389800	1.33505900	-2.51190100	C	2.57446800	-1.01949200	0.40722000
C	-3.52915900	0.69917900	-2.05241800	C	3.51963700	0.10465100	0.30431400
O	-2.20397900	1.99986500	-3.53058300	O	2.86288200	-2.20352000	0.44385400
C	-3.66626900	0.04052500	-0.88396000	C	4.84497000	-0.13172700	0.24401200
C	-4.86729100	-0.61344700	-0.37217800	C	5.92858500	0.83897700	0.14703500
C	-6.05634400	-0.72334000	-1.13033300	C	7.25573500	0.35664700	0.09560700
C	-7.16789100	-1.35479200	-0.59962900	C	8.32470700	1.23576500	0.00525200
C	-7.13019100	-1.90052200	0.70513900	C	8.09677100	2.62755200	-0.03604400
C	-5.96499900	-1.79789100	1.45564400	C	6.79079200	3.11185900	0.01386400
C	-4.84553300	-1.16249700	0.91930400	C	5.72041700	2.22818000	0.10423900
O	-8.22533900	-2.52100000	1.21319400	O	9.14183300	3.48802000	-0.12318500
O	-3.14804100	0.98305700	3.50301700	O	-3.01274700	-6.23381300	-2.45196600
O	8.21192000	-0.94409600	1.08671100	O	-6.49119100	4.44529400	0.34831500
C	7.99370700	0.34774700	1.63230400	C	-7.04678100	3.24071400	0.85152000
O	-8.37770500	-1.53278400	-1.22616300	O	9.65482200	0.89669400	-0.05177800
C	-8.53598600	-1.01884300	-2.54160300	C	9.99870200	-0.48176700	-0.01784200
O	-1.56144500	-1.09746500	3.11391100	O	-1.14805500	-6.48621900	-0.58857600
C	-0.72943900	-2.24173100	2.98087000	C	-0.07106600	-6.64658400	0.32510100
H	-2.49545300	3.18344400	2.26191000	H	-4.34330400	-3.99073300	-2.45647600
H	-0.51054700	3.46426700	0.76409800	H	-3.98808300	-2.09496700	-0.86651900
H	0.60640300	-0.61381100	1.48245100	H	-0.89665900	-4.33262400	1.09667300
H	2.20487000	1.00050000	0.54649400	H	-1.75677100	-2.33935600	2.10472800
H	0.91775100	-0.03509400	-1.22049000	H	-0.99809000	-0.14513800	1.48652400
H	2.64565100	2.18619400	-2.40225400	H	-1.75449300	-0.88003400	-1.37252500
H	2.27272400	0.62576600	-3.13625000	H	-0.47955500	0.28035700	-1.02465300
H	-0.01045200	2.77540800	-1.89513400	H	0.33604600	-2.24077700	-0.29896300
H	0.14268000	1.56583600	-3.16967800	H	0.49417500	-2.19425200	1.45839000
H	4.15812100	1.12472400	-0.75888000	H	-3.39520200	0.67929600	-0.11440500
H	3.73060300	-1.20266700	-2.69848700	H	-1.18073400	2.48570700	-1.21845200
H	5.14740100	-3.04901700	-2.59303100	H	-1.65869900	4.73908900	-1.63365500

H	7.14151300	-4.25211900	-1.70959100	H	-3.14841900	6.73281200	-1.52555600
H	5.99049400	0.25861200	0.02189100	H	-4.73921900	2.34397900	0.16785500
H	9.17309200	-2.75652400	0.78061100	H	-6.41251000	6.45274800	-0.16982700
H	3.37954800	2.86278700	1.30564700	H	-4.55851400	-2.80730700	1.85778600
H	1.84341000	3.44193700	1.97464800	H	-3.74460800	-2.09639400	3.26470200
H	2.00372400	5.51776700	0.56955400	H	-5.95080900	-0.73692400	1.60295600
H	3.52777700	4.93726100	-0.12267300	H	-6.15157900	-1.42925400	3.22381300
H	3.43510700	5.34558200	1.60394300	H	-5.12013900	-0.00215200	2.98905100
H	-4.35098500	0.83128800	-2.74865600	H	3.09207500	1.10115000	0.28465800
H	-2.79504300	-0.01131700	-0.23556800	H	5.14187200	-1.17933500	0.27297100
H	-6.09214200	-0.31636500	-2.13319500	H	7.42629600	-0.71365900	0.12806600
H	-5.95282600	-2.21947100	2.45501200	H	6.63587500	4.18493800	-0.01904500
H	-3.94422300	-1.07903700	1.51798300	H	4.71217100	2.62591300	0.14268400
H	-8.91694600	-2.47852200	0.53369500	H	9.95136000	2.95314800	-0.14488200
H	-3.10846400	0.08254200	3.86001900	H	-2.37188800	-6.92318000	-2.21781800
H	8.03672100	1.12123400	0.85570700	H	-8.05343400	3.48766400	1.19194500
H	8.79529400	0.51384900	2.35319400	H	-6.46273400	2.85258800	1.69519200
H	7.02604500	0.40604200	2.14595100	H	-7.10516100	2.47139500	0.07156400
H	-8.39916100	0.06902700	-2.56271800	H	11.08696400	-0.52501800	-0.07335700
H	-9.55577400	-1.26178900	-2.84221400	H	9.56892600	-1.02007500	-0.87125300
H	-7.82986200	-1.48594700	-3.23870900	H	9.66260600	-0.95269700	0.91384400
H	-0.65836700	-2.56335000	1.93496100	H	0.36440600	-7.62378900	0.11288400
H	-1.19924300	-3.03002900	3.57027500	H	0.68907800	-5.86878900	0.18521100
H	0.27808000	-2.04828100	3.36867300	H	-0.42306700	-6.62625700	1.36389300

Part 2. Supplementary Figures

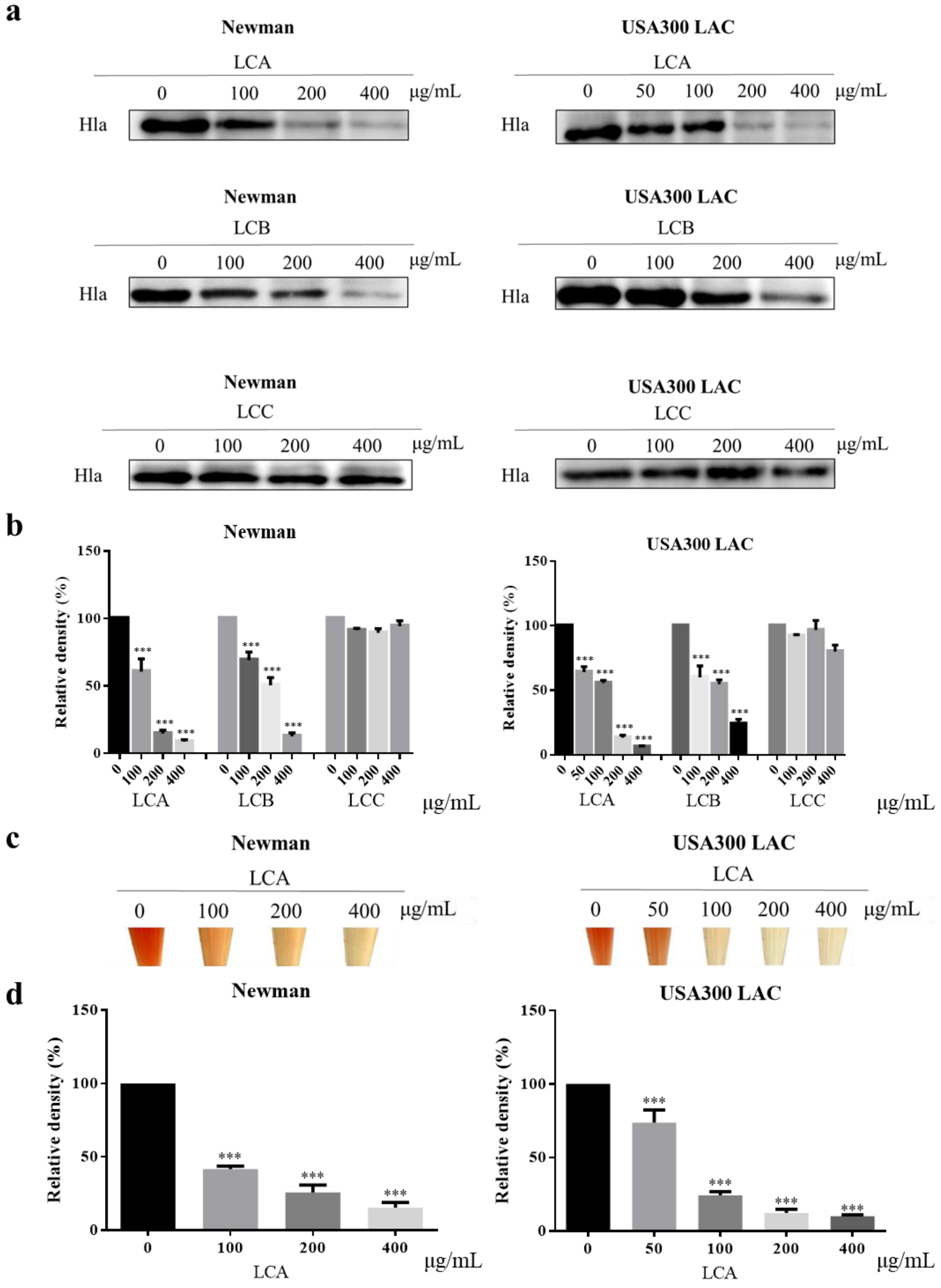

Figure S3. LCA inhibits the expression of Hla in both Newman and USA300 LAC strain. **a**. Effect of three chuanxiong extracts on the production of Hla in Newman and USA300 LAC strain. **b**. The statistical analysis of western blotting assay of three chuanxiong extracts. Relative band density was analyzed using Image J. All experiments were performed in triplicate. **c**. Hemolysis assay in Newman and USA300 LAC strain treated with different concentrations of LCA. **d**. The statistical analysis of hemolysis assay of LCA. The results are shown as the standard error of the mean; all experiments were performed in triplicate. ***$P < 0.001$, in comparison with control, one-way ANOVA.

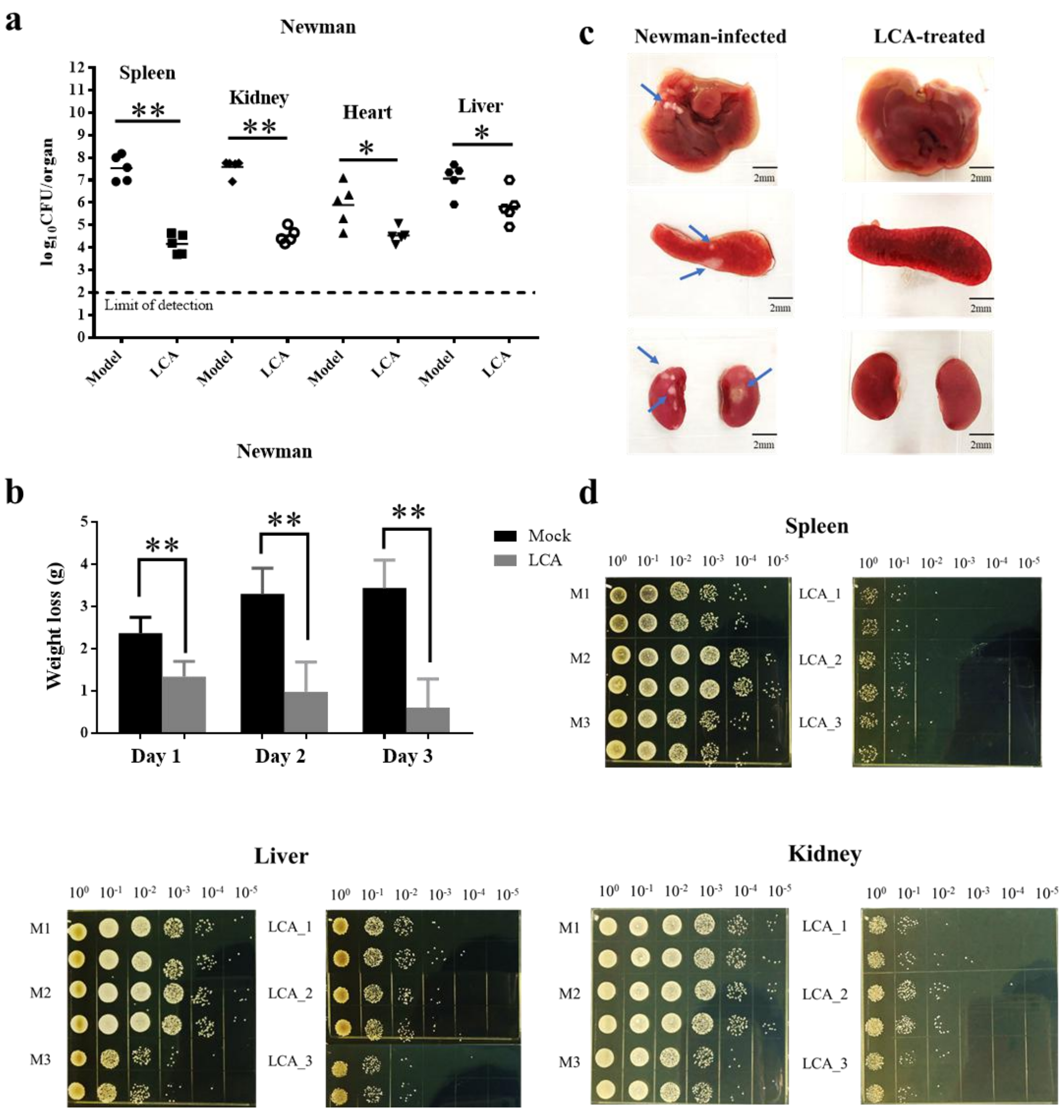

Figure S4. Effect of LCA (100 mg/kg/d) on the survival of the *S. aureus* Newman strain in the spleens, livers, kidneys and hearts of mice (n = 5) intraperitoneally challenged with 1 × 10^8 CFU of bacteria. **a**. Statistical analysis for the enumeration of colony forming units (CFU) is displayed. *P < 0.05, **P < 0.01, in comparison with control, Mann-Whitney test, two-tailed. Each symbol represents the value for an individual mouse. Horizontal bars indicate the observational means and the dashed line marks limit of detection. **b**. Weight loss of Newman-infected mice treated with LCA in the day 1, 2 and 3. The results are shown as the standard error of the mean; **P < 0.01, in comparison with control, two tailed *t*-test. **c**. Representative photographs of Newman-infected mice liver, spleen and kidney treated with or without LCA. **d**. Representative photographs of the TSA plates for CFU enumeration of the mice spleens, livers and kidneys infected by Newman with or without treatment of LCA.

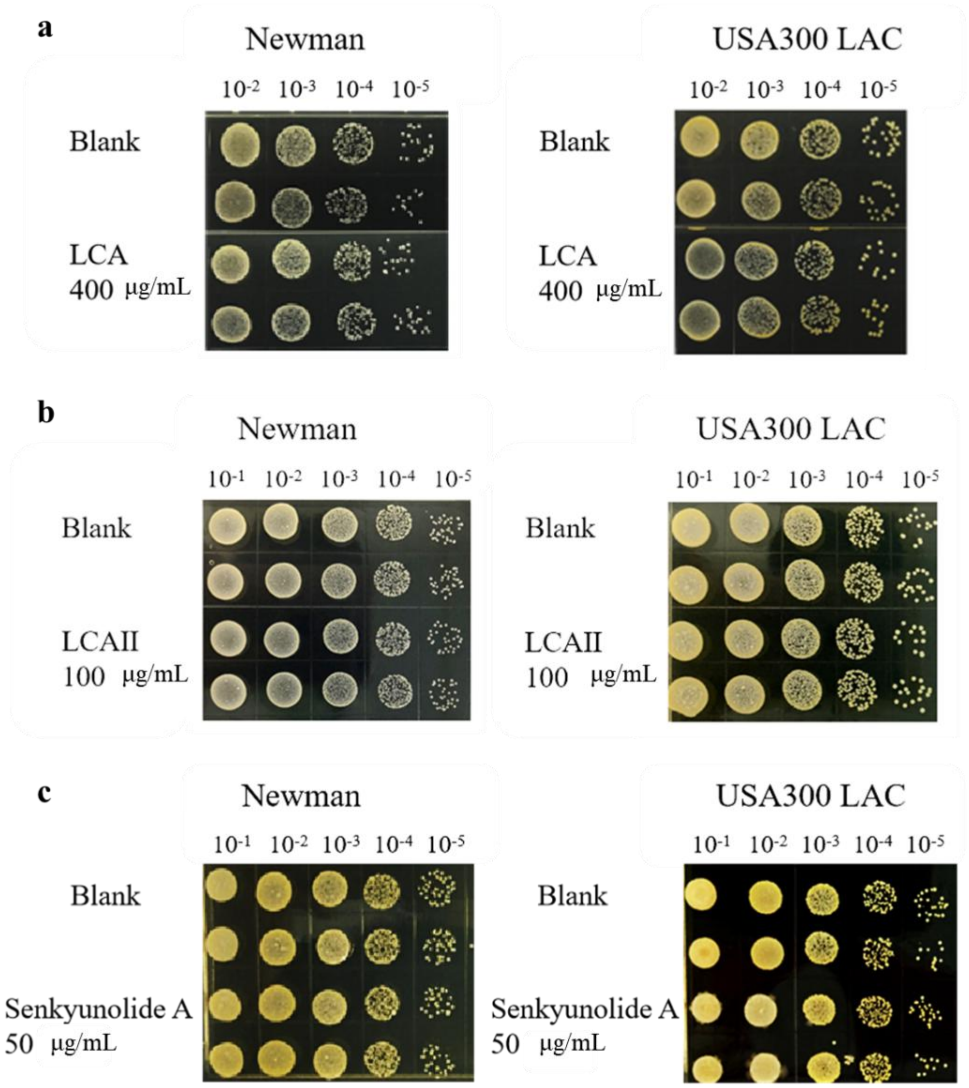

Figure S5. LCA, LCAII and senkyunolide A have no toxic effect on the growth of *S. aureus*. Mid-log bacteria culture (OD_{600} = 0.3) treated with LCA, LCAII and senkyunolide A was quantified by the plating method after co-incubation. The culture with 1% DMSO treatment served as control. The bacteriostatic activity was determined after comparison with the colony forming unit (CFU) of the control. **a**. LCA (400 μg/mL) did not affect the growth of *S. aureus* USA300 LAC strain and Newman strain. **b**. LCAII (100 μg/mL) did not affect the growth of *S. aureus* Newman strain and USA300 LAC strain. **c**. Senkyunolide A (50 μg/mL) would not affect the growth of *S. aureus* Newman strain and USA300 LAC strain.

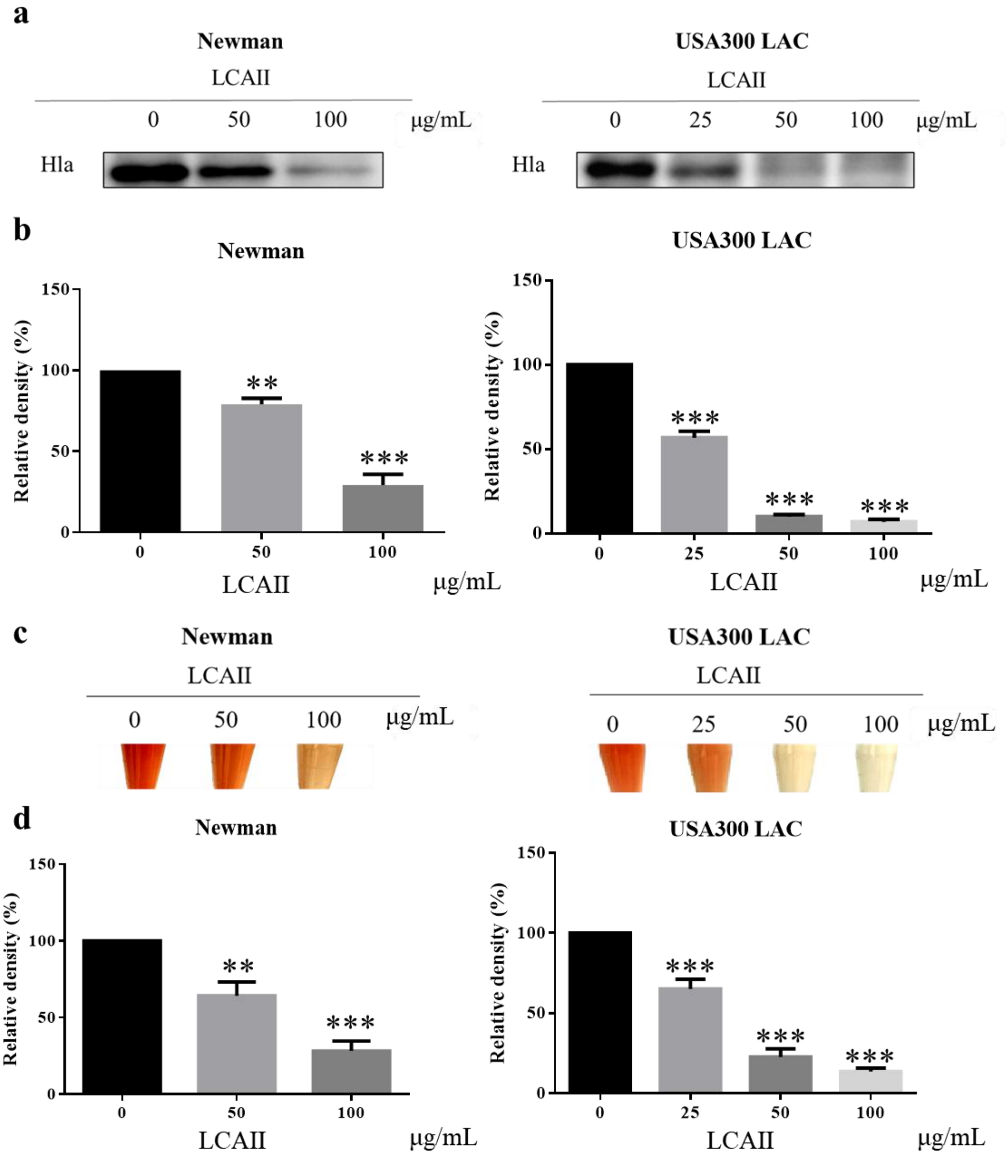

Figure S6. LCAII shows substantial inhibition against Hla in both Newman and USA300 LAC strain. **a**. Western blotting analysis of Hla expression in Newman and USA300 LAC strain treated with different concentrations of LCAII. **b**. The statistical analysis of western blotting assay of LCAII. Relative band density was analyzed using Image J. All experiments were performed in triplicate. **c**. Hemolysis assay in Newman and USA300 LAC strain treated with different concentrations of LCAII. d. The statistical analysis of hemolysis assay of LCAII. The results are shown as the standard error of the mean; all experiments were performed in triplicate. **P < 0.01 and ***P < 0.001, in comparison with control, one-way ANOVA.

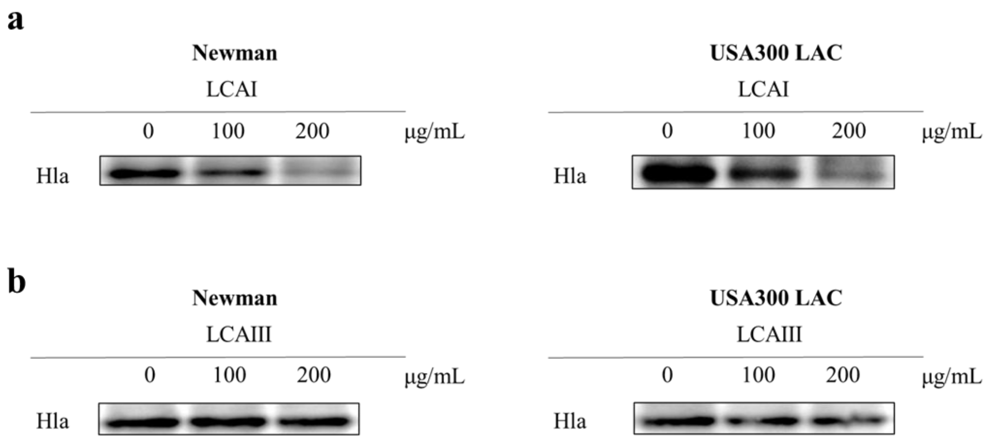

Figure S7. LCAI and LCAIII show weak inhibitory effect on Hla expression. **a**. Western blotting analysis of Hla expression treated with different concentrations of LCAI in Newman strain and USA300 LAC strain. **b**. Western blotting analysis of Hla expression treated with different concentrations of LCAIII in Newman strain and USA300 LAC strain.

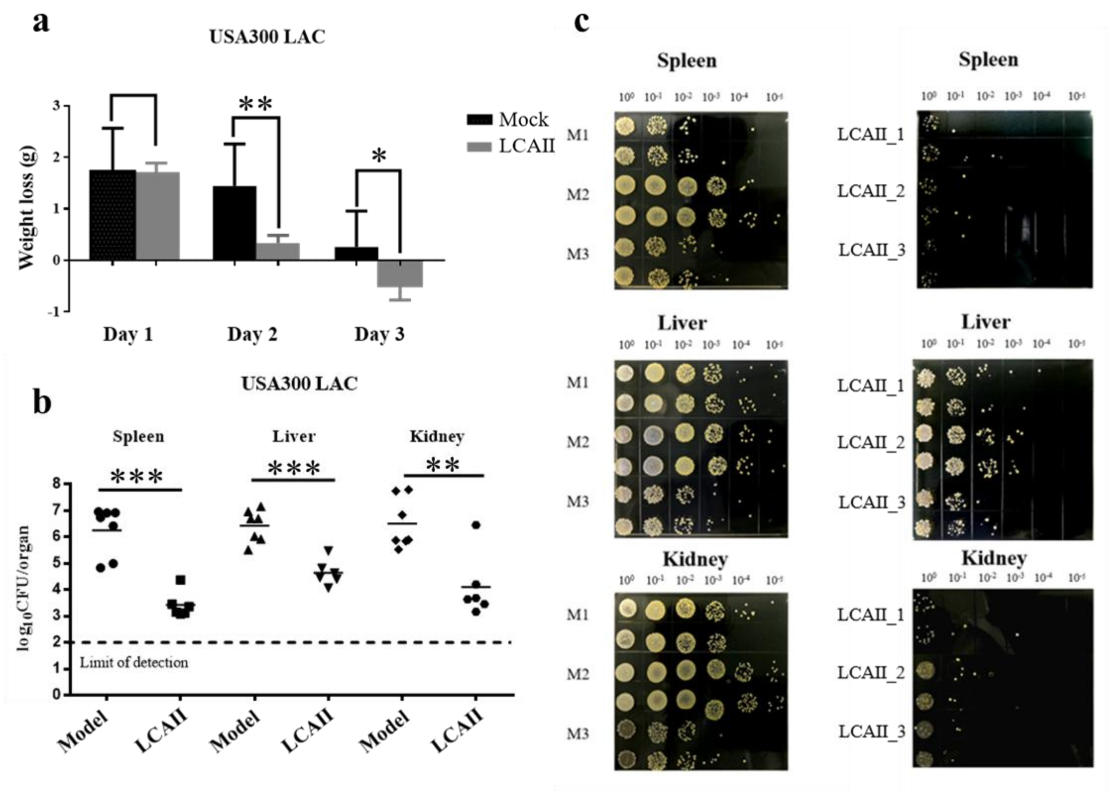

Figure S8. Effect of LCAII (100 mg/kg/d) on the survival of the *S. aureus* USA300 LAC strain in the spleens, livers and kidneys of mice (n = 7) intraperitoneally challenged with 7.4 × 10^8 CFU of bacteria. **a**. Weight loss of USA300-infected mice treated with LCAII in the day 1, 2 and 3. The results are shown as the standard error of the mean; *P < 0.05; **P < 0.01, in comparison with control, two tailed *t*-test. **b**. Statistic analysis for the enumeration of colony forming units (CFU) is displayed. **P < 0.01, ***P < 0.001, in comparison with control, Mann-Whitney test, two-tailed. Each symbol represents the value for an individual mouse. Horizontal bars indicate the observational means and the dashed line marks limit of detection; **c**. Representative photographs of the TSA plates for the CFU enumeration of the mice spleens, livers and kidneys infected by USA300 LAC with or without treatment of LCAII.

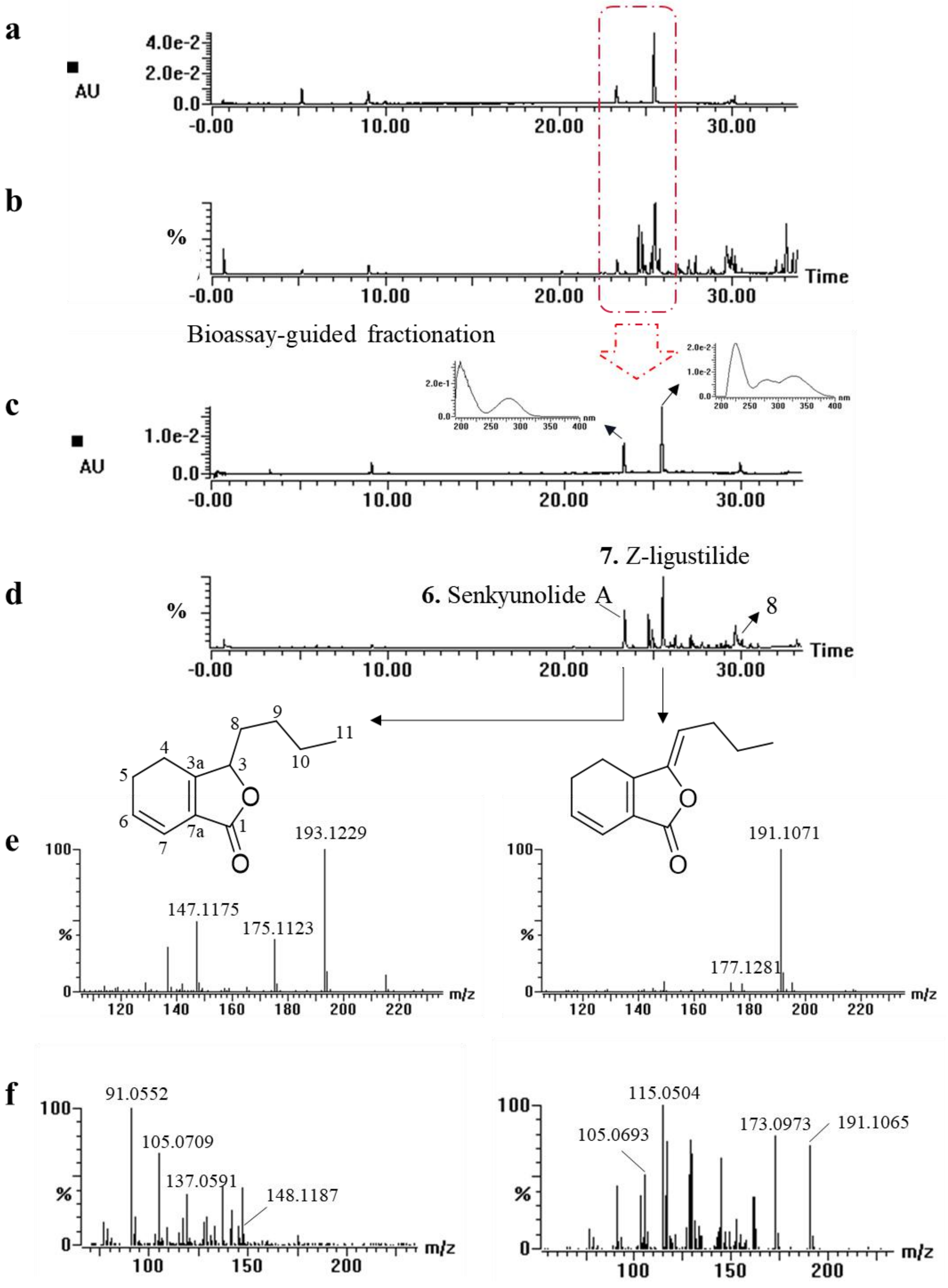

Figure S9. Identification of two main constituents in LCAII by UPLC-PDA-QTOF-MS. **a**. UV chromatogram of LCA at 280 nm. **b**. Base peak chromatogram (BPC) of LCA. **c**. UV chromatogram of LCAII at 280 nm. **d**. Base peak chromatograms (BPC) of LCAII. **e**. Structures and MS spectra of two main constituents in LCAII. **f**. MS/MS spectra of senkyunolide A at *m/z* 193.1229 and Z-ligustilide at *m/z* 191.1072.

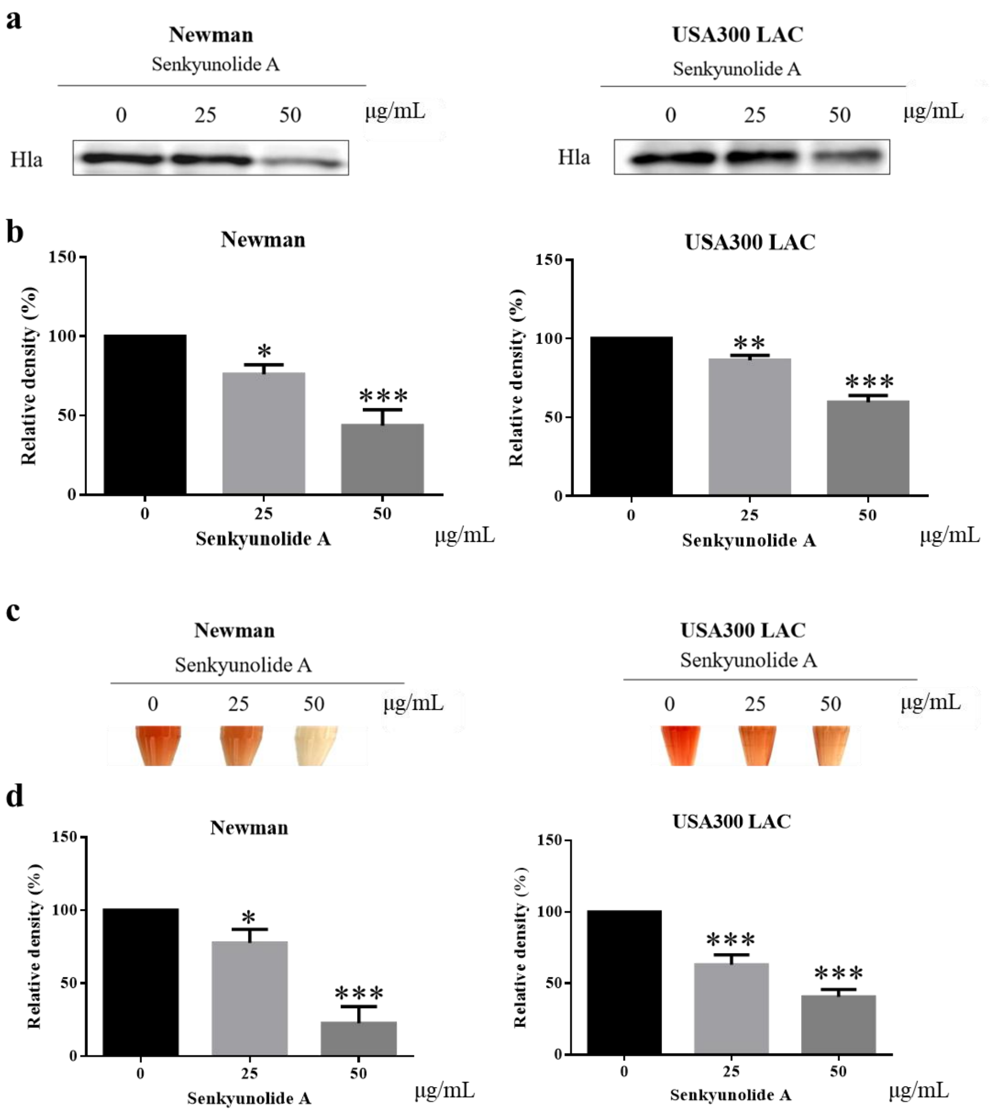

Figure S10. Senkyunolide A reduces hemolytic activity of *S. aureus* by inhibiting Hla production. **a**. Western blotting analysis of Hla expression treated with different concentrations of senkyunolide A in Newman and USA300 LAC strain. **b**. The statistical analysis of western blotting assay of senkyunolide A. Relative band density was analyzed using Image J. All experiments were performed in triplicate. **c**. Hemolysis assay treated with different concentrations of senkyunolide A in Newman and USA300 LAC strain. **d**. The statistical analysis of hemolysis assay of senkyunolide A. The results are shown as the standard error of the mean; all experiments were performed in triplicate. *P < 0.05; **P < 0.01 and ***P < 0.001, in comparison with control, one-way ANOVA.

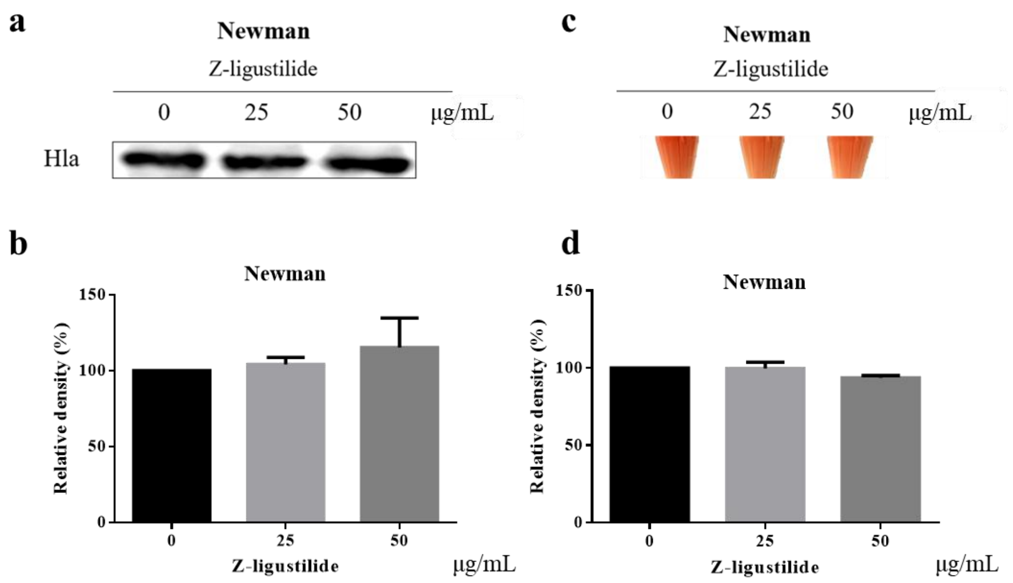

Figure S11. *Z*-ligustilide has no effect on Hla expression. **a**. Western blotting analysis of Hla expression treated with different concentrations of *Z*-ligustilide in Newman strain. **b**. The statistical analysis of western blotting assay of *Z*-ligustilide. Relative band density was analyzed using Image J. All experiments were performed in triplicate. **c**. Hemolysis assay treated with different concentrations of *Z*-ligustilide in Newman strain. **d**. The statistical analysis of hemolysis assay of *Z*-ligustilide. The results are shown as the standard error of the mean; all experiments were performed in triplicate. One-way ANOVA.

a

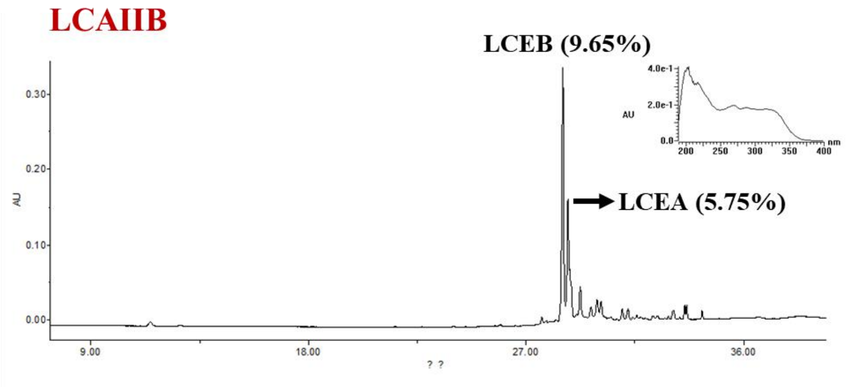

b

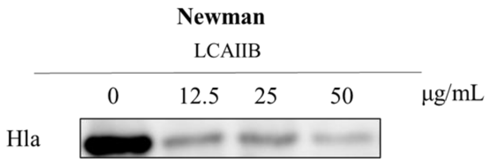

Figure S12. LCAIIB shows outstanding inhibition against Hla in Newman strain. **a**. UV chromatogram of LCAIIB at 280 nm. **b**. Western blotting analysis of Hla expression treated with different concentrations of LCAIIB in Newman strain.

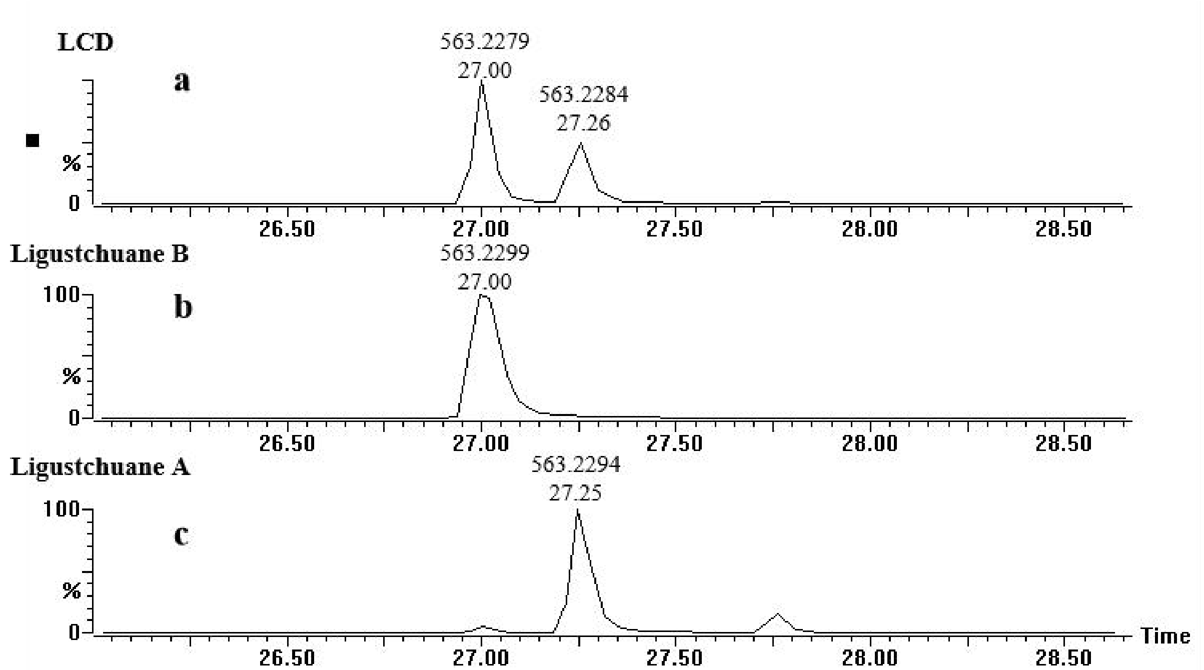

Figure S13. UPLC-ESI-QTOF-MS analysis of compounds **1** and **2** in a new plant extract of chuanxiong. **a**. the extracted ion chromatogram (EIC) of LCD (ions at *m/z* 563.228). **b**. the extracted ion chromatogram (EIC) of compound **2** (ions at *m/z* 563.228). **c**. EIC of compound **1** (ions at *m/z* 563.228).

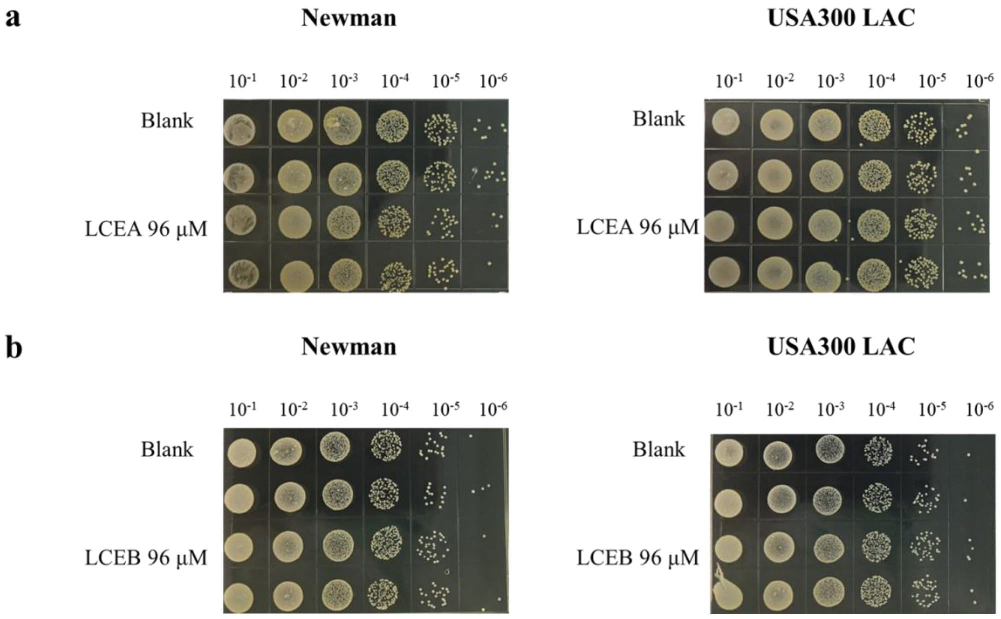

Figure S14. Ligustchuanes A (**1**) and B (**2**) have no toxic effect on the growth of *S. aureus*. Mid-log bacteria culture (OD_{600} = 0.3) treated with ligustchuanes A (**1**) and B (**2**) was quantified by the plating method after co-incubation. The culture with 1% DMSO treatment served as control. The bacteriostatic activity was determined after comparison with the colony forming unit (CFU) of the control. **a**. Ligustchuane A (96 *μ*M) did not affect the growth of *S. aureus* Newman strain and USA300 LAC strain. **b**. Ligustchuane B (96 *μ*M) did not affect the growth of *S. aureus* Newman strain and USA300 LAC strain.

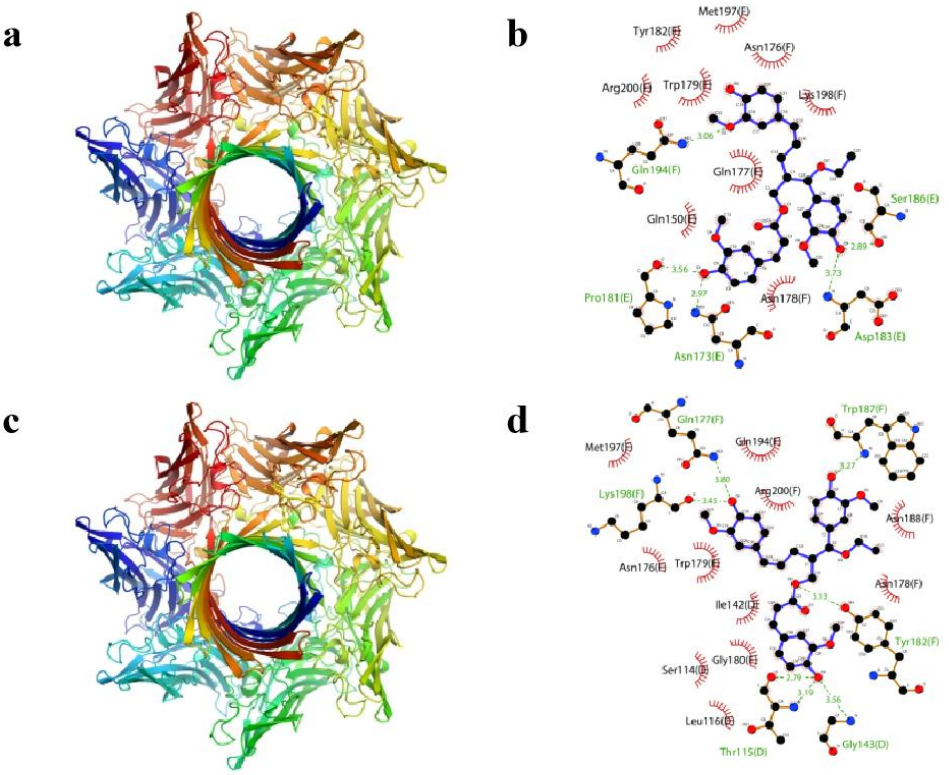

Figure S15. Compounds (±) **2** may interact with *α*-hemolysin heptamer of *S. aureus*. The proposed 3D (**a**) and 2D (**b**) binding models of (−) **2** with *α*-hemolysin heptamer. The proposed 3D (**c**) and 2D (**d**) binding models of (+) **2** with *α*-hemolysin heptamer. The green dashed lines represent hydrogen bonds.

Part 3. HRESIMS, IR, ECD, and NMR spectra of compounds **1** and **2**

Ligustchuane A (**1**)

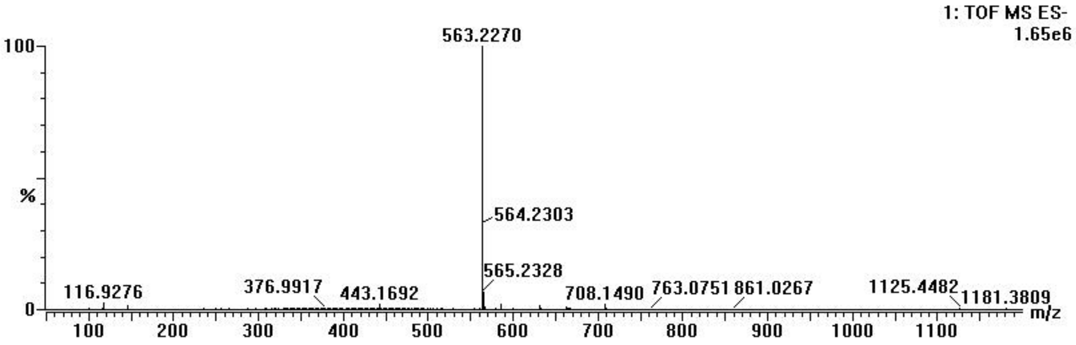

Figure S16. HRESIMS spectrum of **1**.

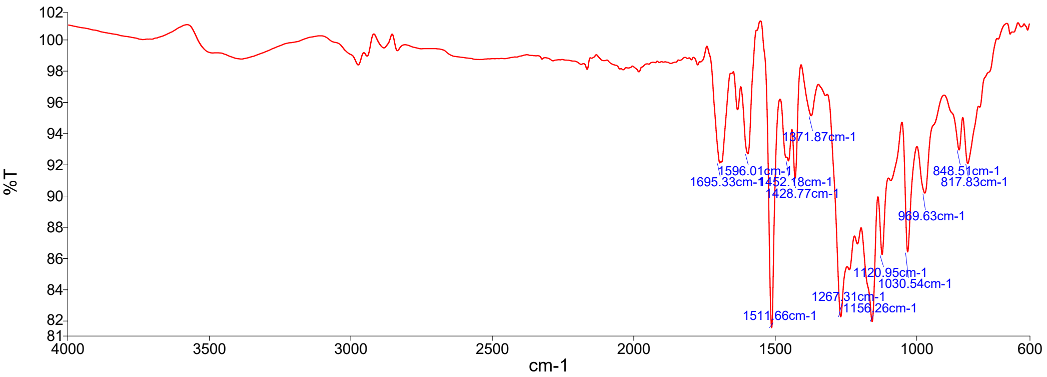

Figure S17. IR (KBr, disc) spectrum of **1**.

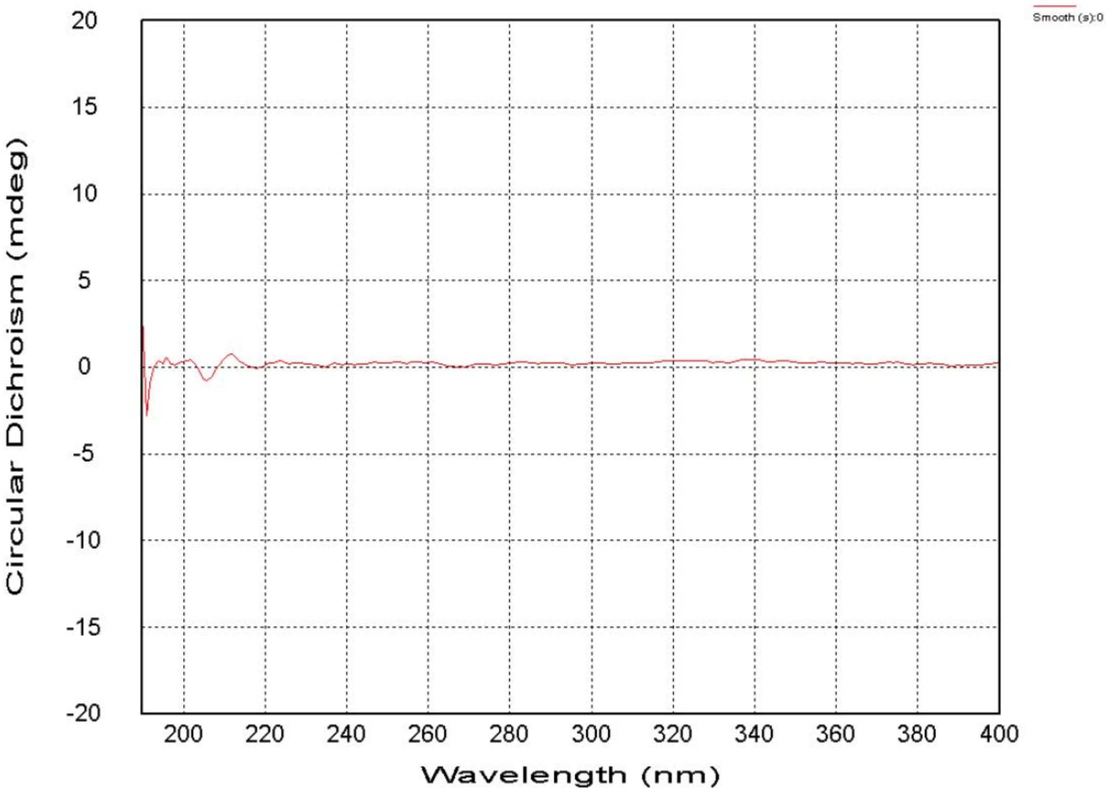

Figure S18. Experimental ECD spectrum of **1**.

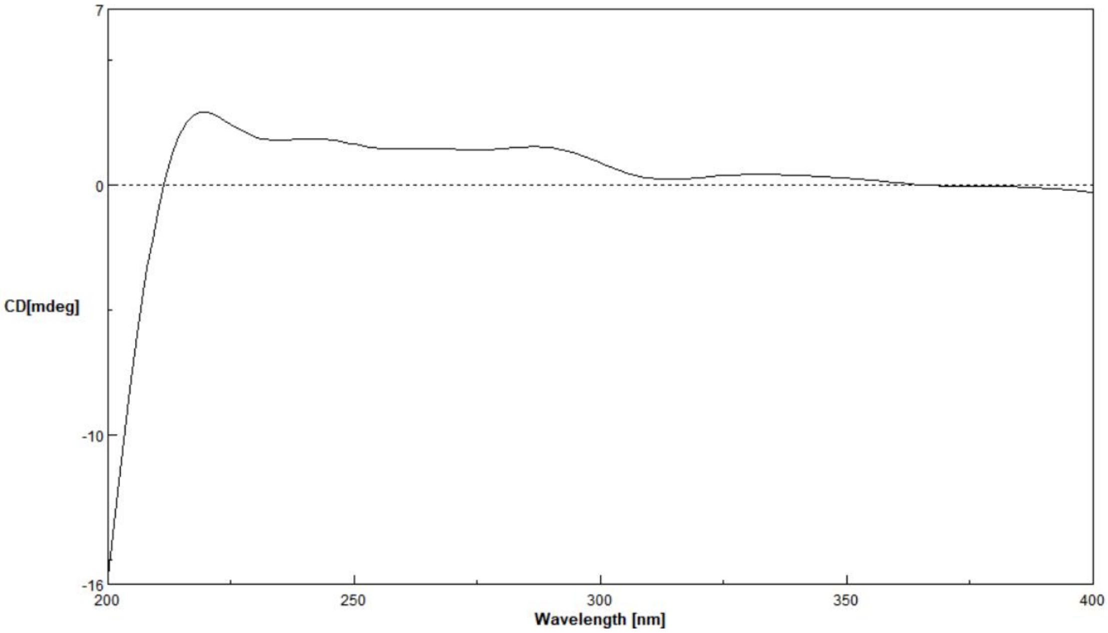

Figure S19. Experimental ECD spectrum of (+) **1**.

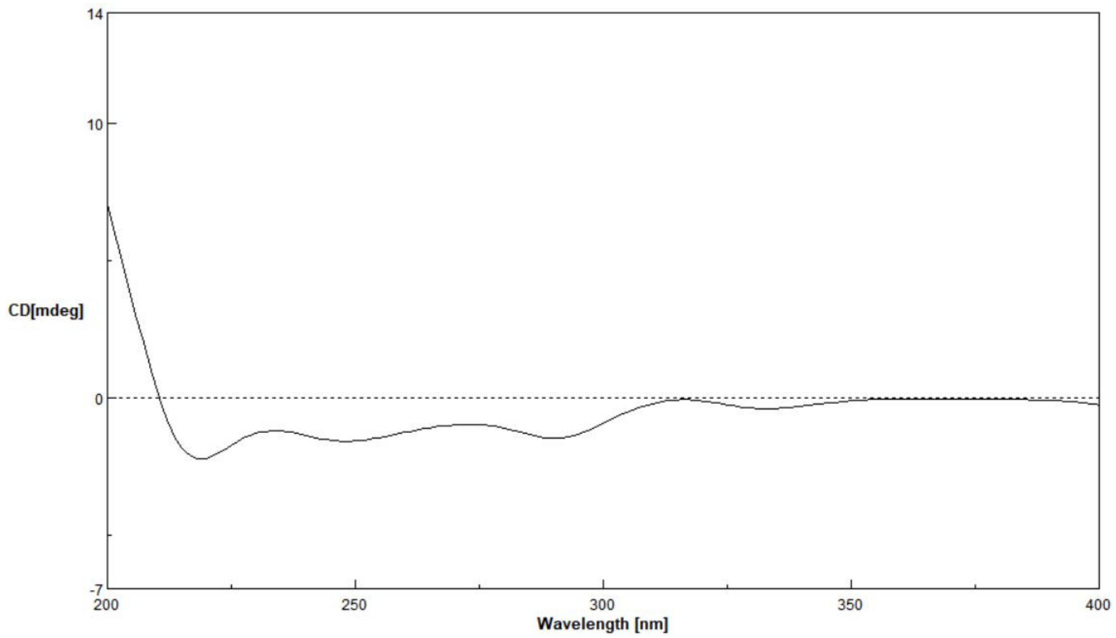

Figure S20. Experimental ECD spectrum of (-) **1**.

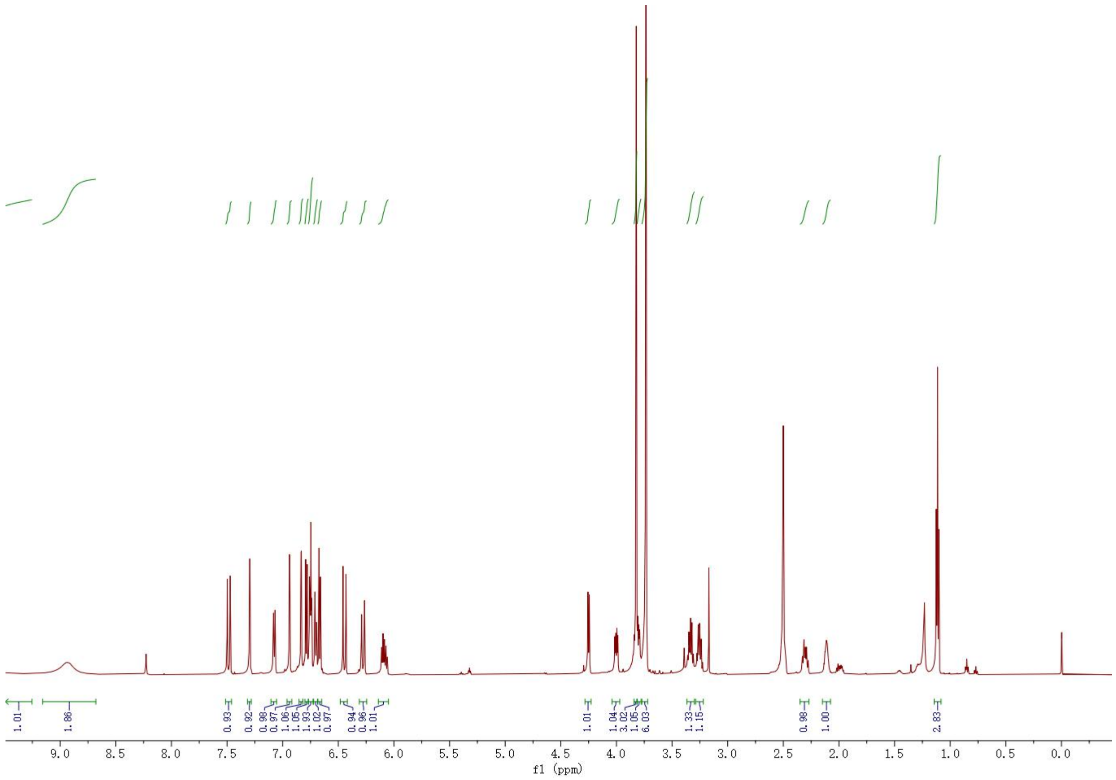

Figure S21. ^{1}H NMR spectrum (DMSO-d_6, 600 MH_Z) of **1**.

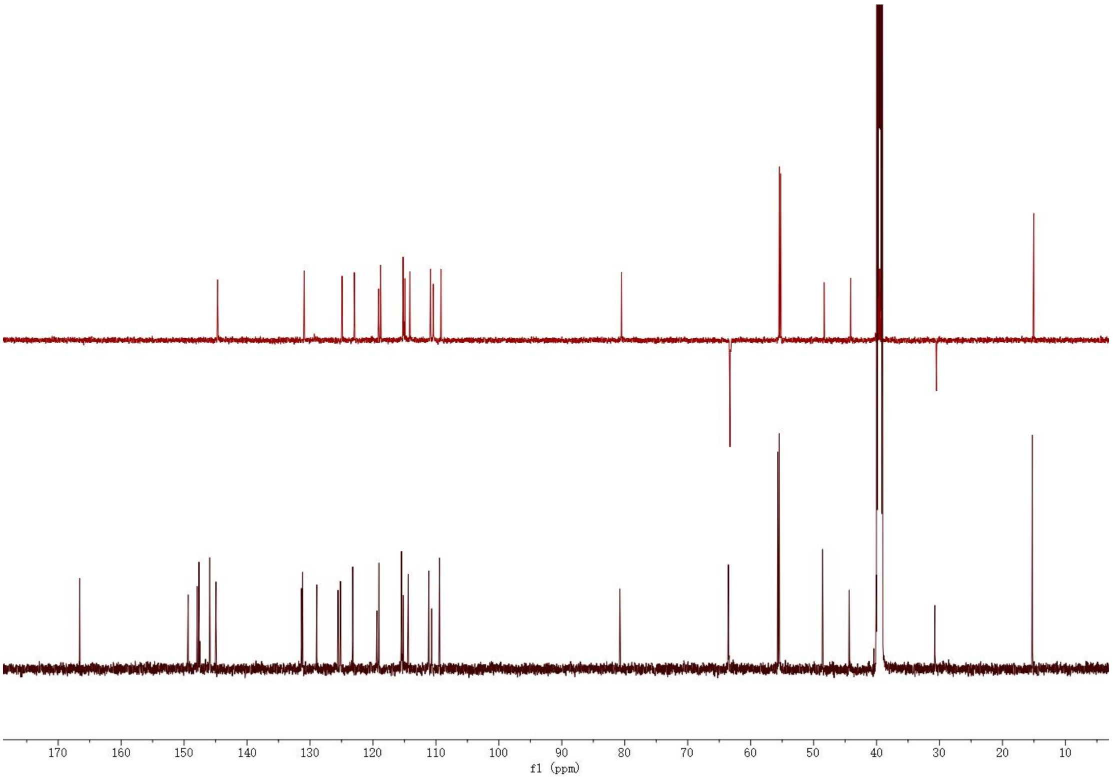

Figure S22. ^{13}C NMR and DEPT-135 spectra (DMSO-d_6, 150 MHz) of **1**.

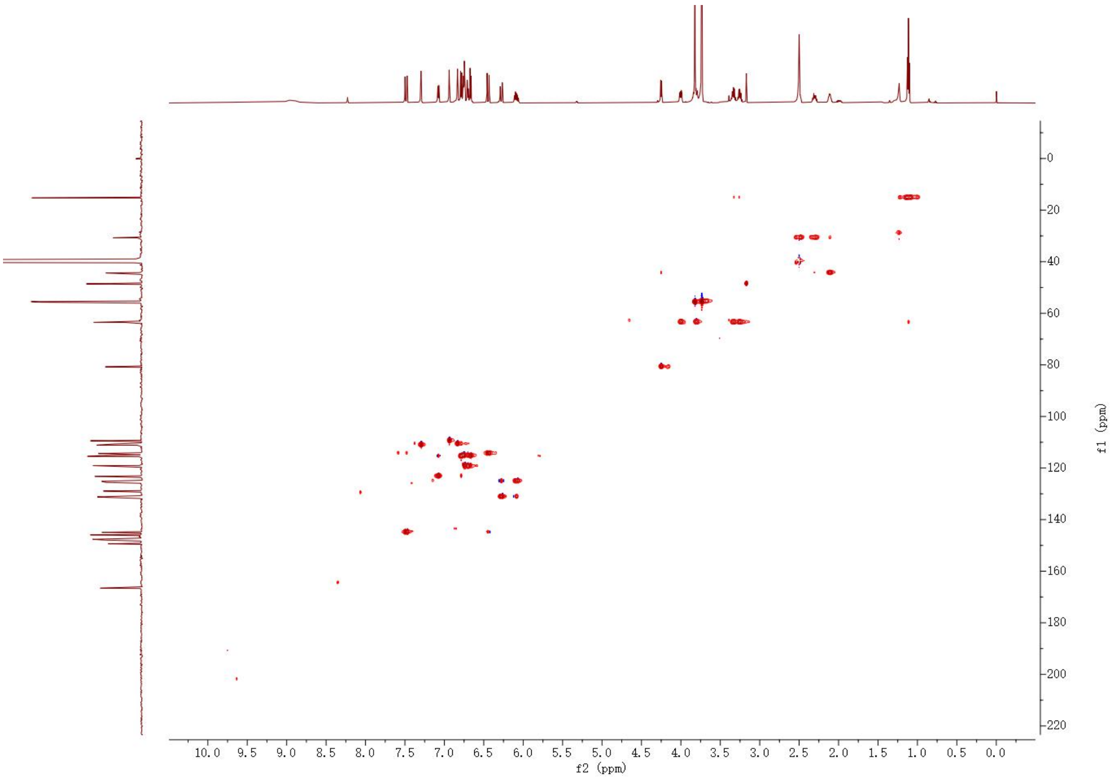

Figure S23. HSQC NMR spectrum (DMSO-d_6, 600 MHz, 150 MHz) of **1**.

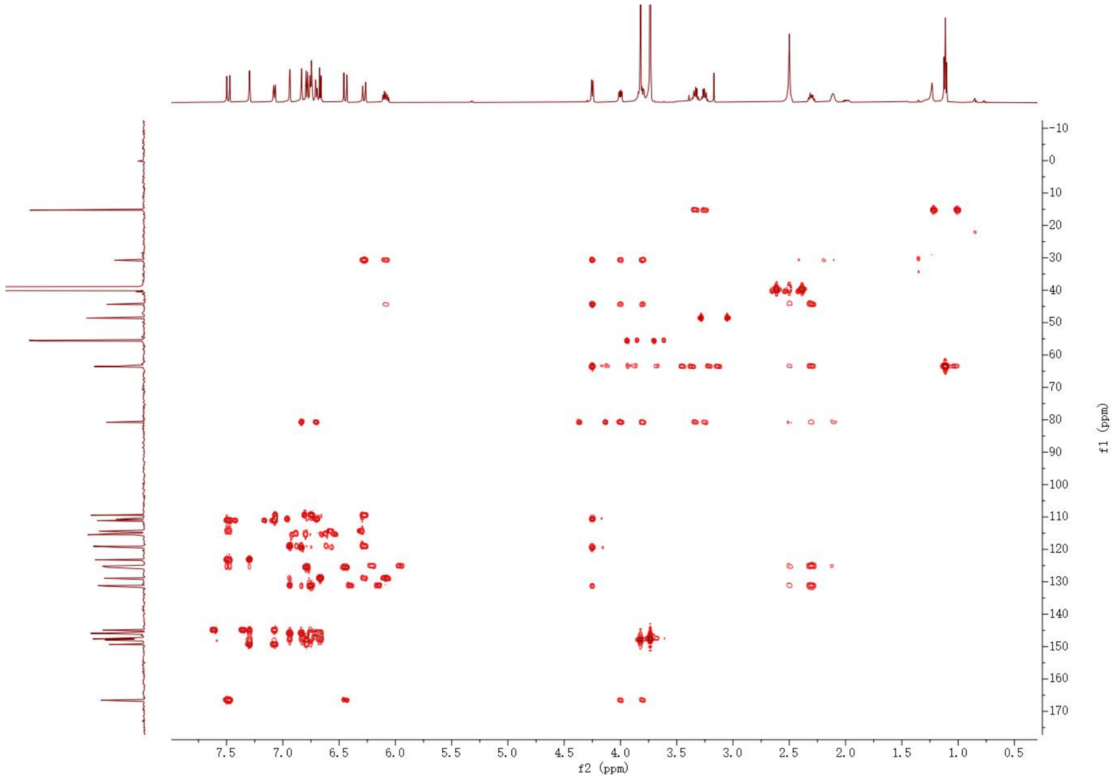

Figure S24. HMBC NMR spectrum (DMSO-d_6, 600 MHz, 150 MHz) of **1**.

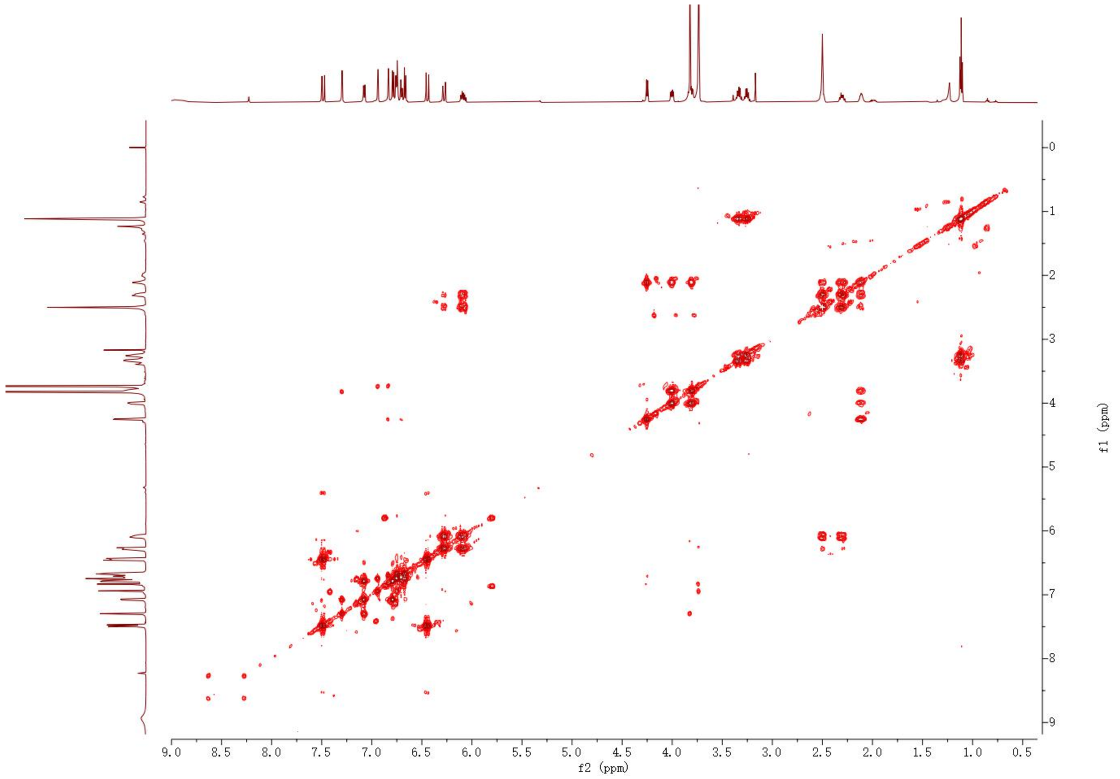

Figure S25. ^{1}H-^{1}H COSY NMR spectrum (DMSO-d_6, 600 MHz) of **1**.

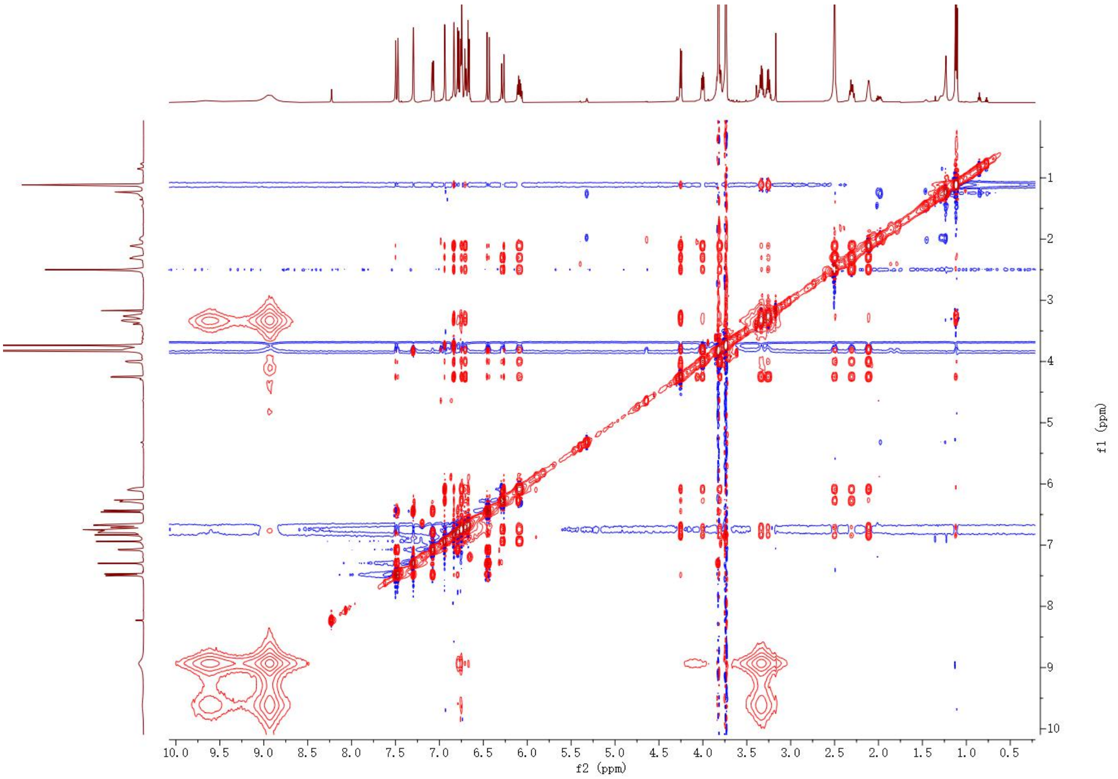

Figure S26. NOESY NMR spectrum (DMSO-d_6, 600 MHz) of **1**.

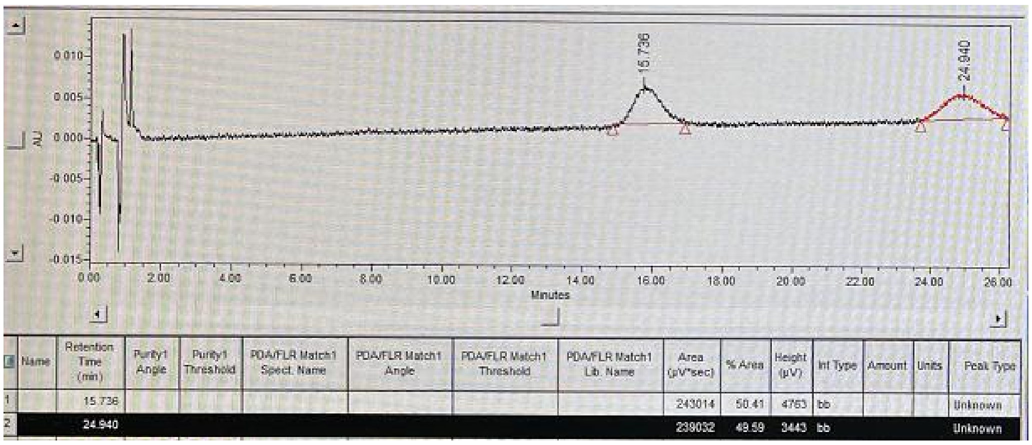

Name	Retention Time (min)	Purity1 Angle	Purity1 Threshold	PDA/FLR Match1 Spect. Name	PDA/FLR Match1 Angle	PDA/FLR Match1 Threshold	PDA/FLR Match1 Lib. Name	Area (µV*sec)	% Area	Height (µV)	Int Type	Amount	Units	Peak Type
1	15.736							243014	50.41	4763	bb			Unknown
2	24.940							239032	49.59	3443	bb			Unknown

Figure S27. Enantioseparation via chiral-phase UPC^2 on a Daicel Chiralpak IG column of **1**.

Ligustchuane B (**2**)

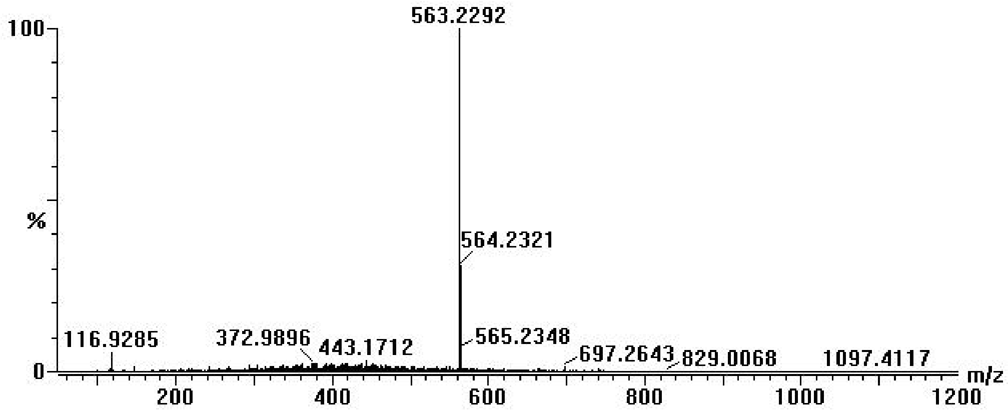

Figure S28. HRESIMS spectrum of **2**.

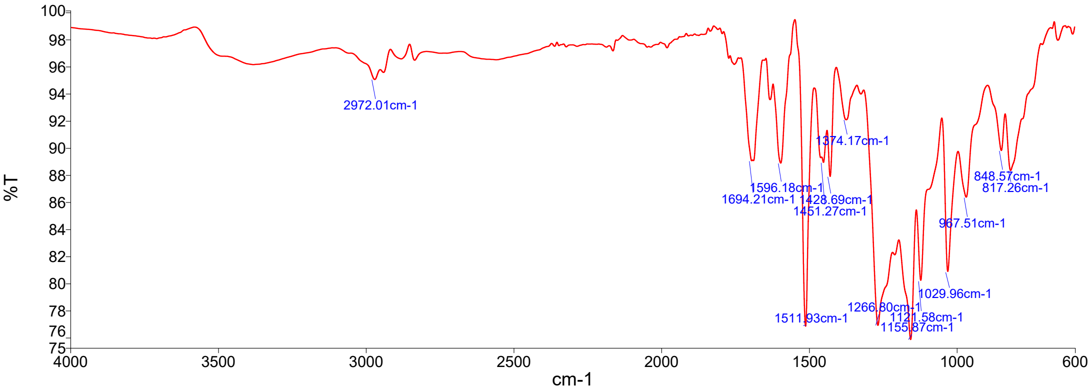

Figure S29. IR (KBr, disc) spectrum of **2**.

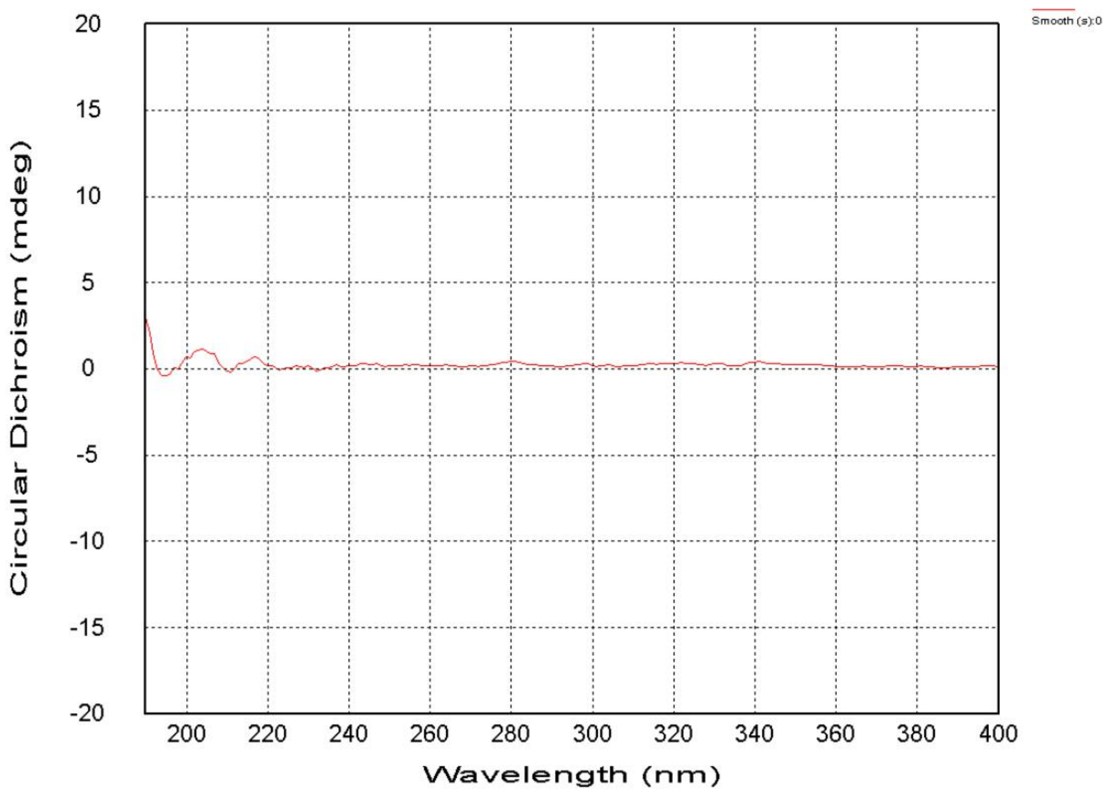

Figure S30. Experimental ECD spectrum of **2**.

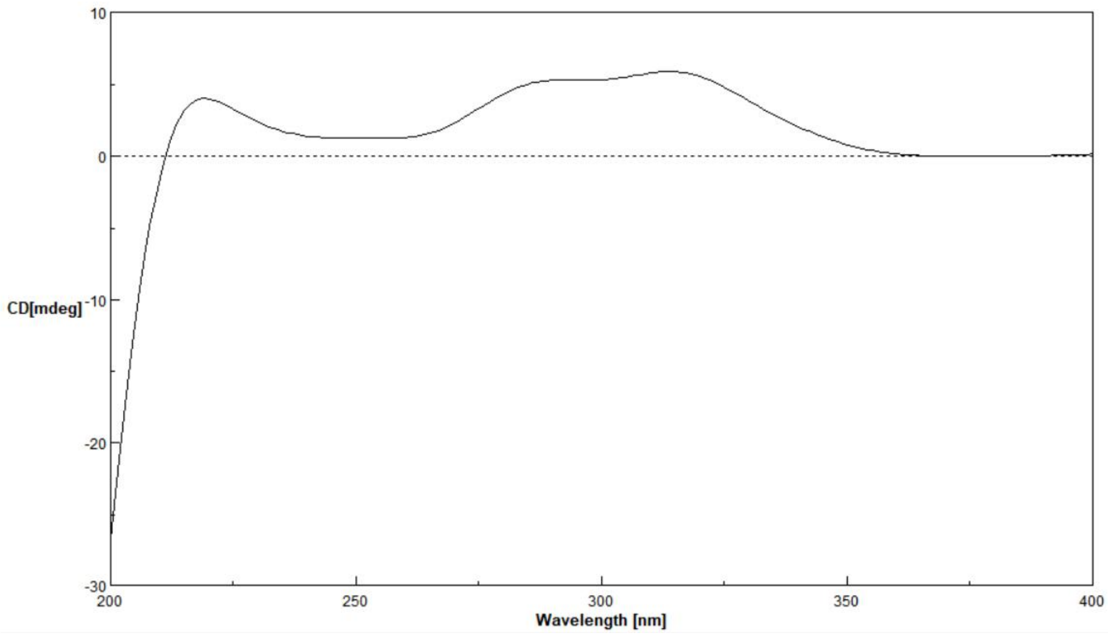

Figure S31. Experimental ECD spectrum of (+) **2**.

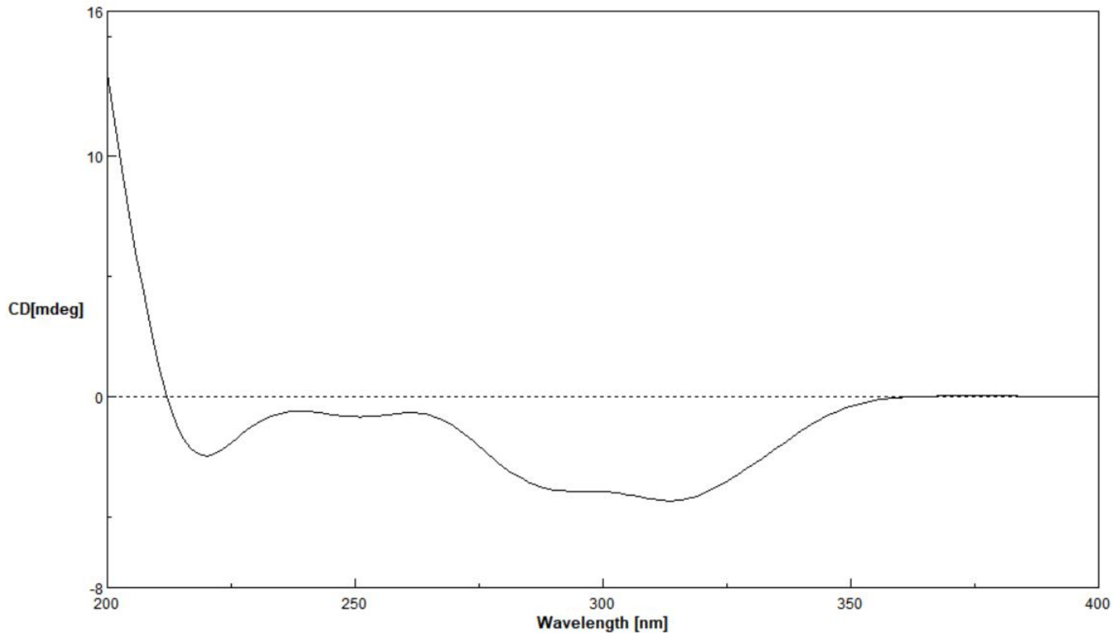

Figure S32. Experimental ECD spectrum of (-) **2**.

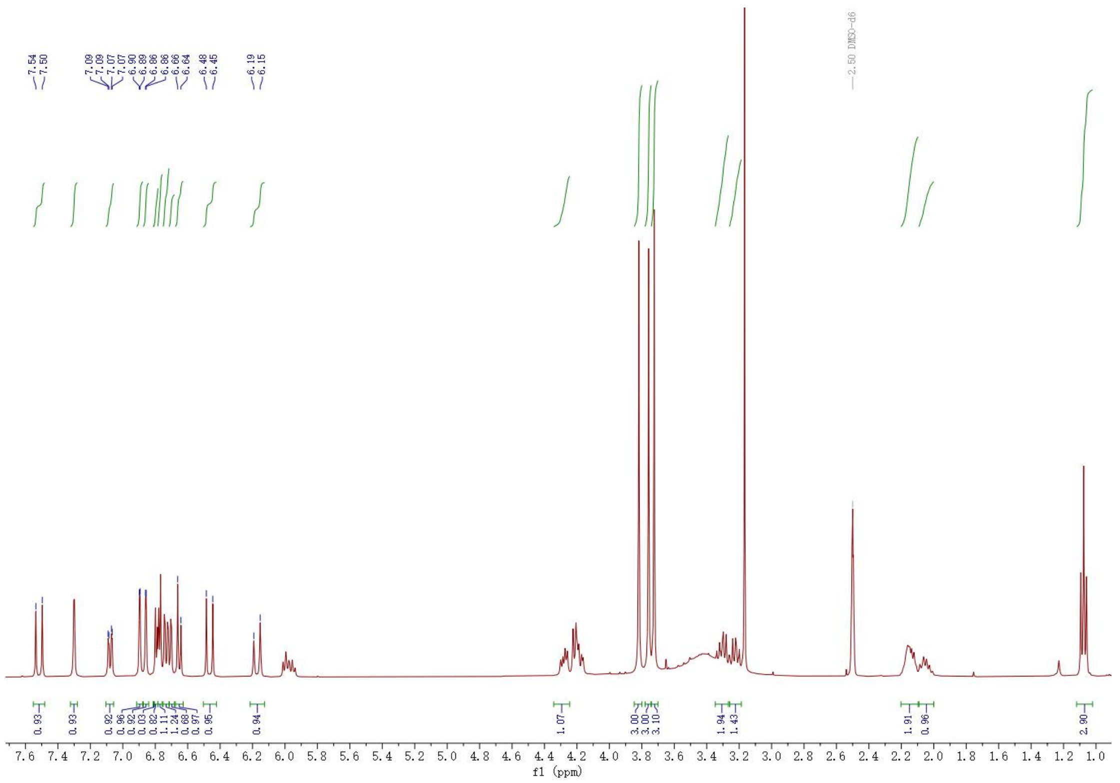

Figure S33. ^{1}H NMR spectrum (DMSO-d_6, 400 MHz) of **2**.

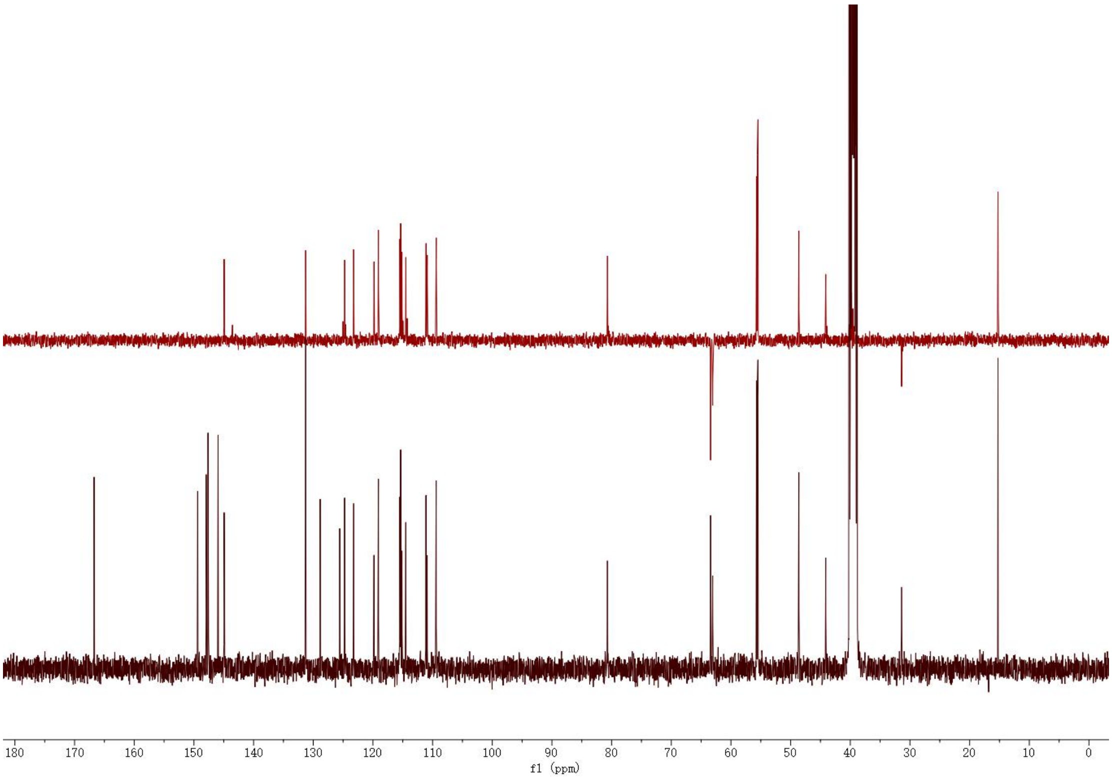

Figure S34. ^{13}C NMR and DEPT-135 spectra (DMSO-d_6, 100 MHz) of **2**.

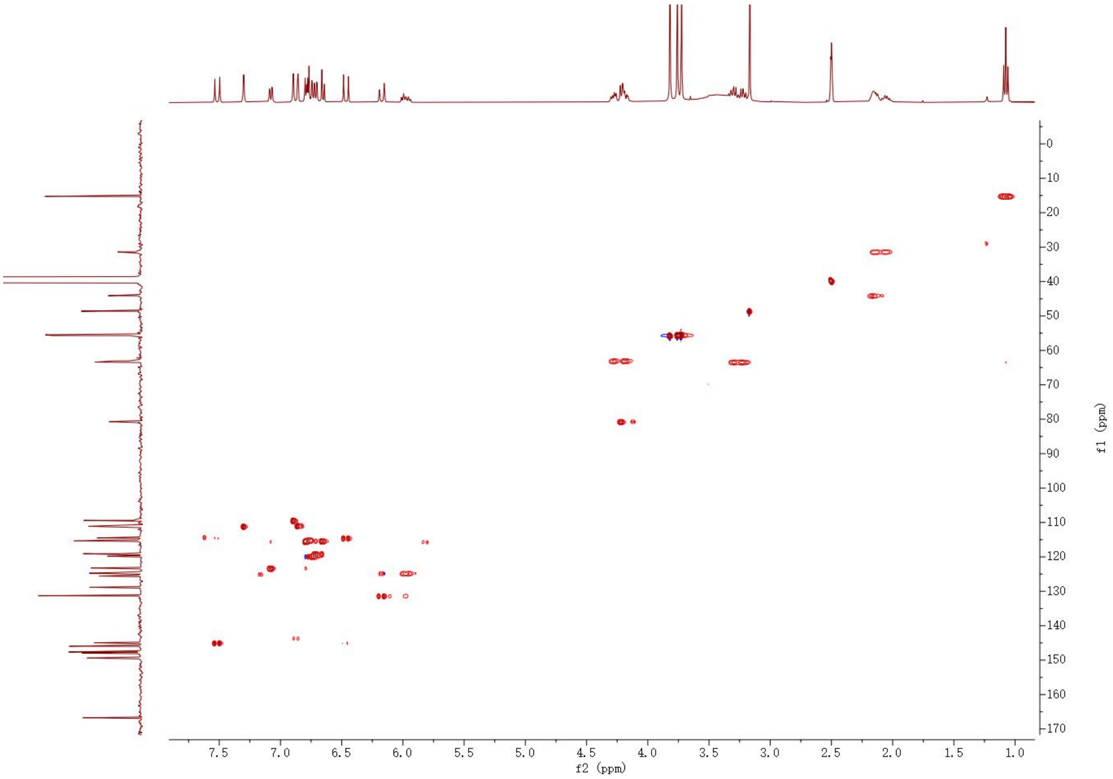

Figure S35. HSQC NMR spectrum (DMSO-d_6, 400 MHz, 100 MHz) of **2**.

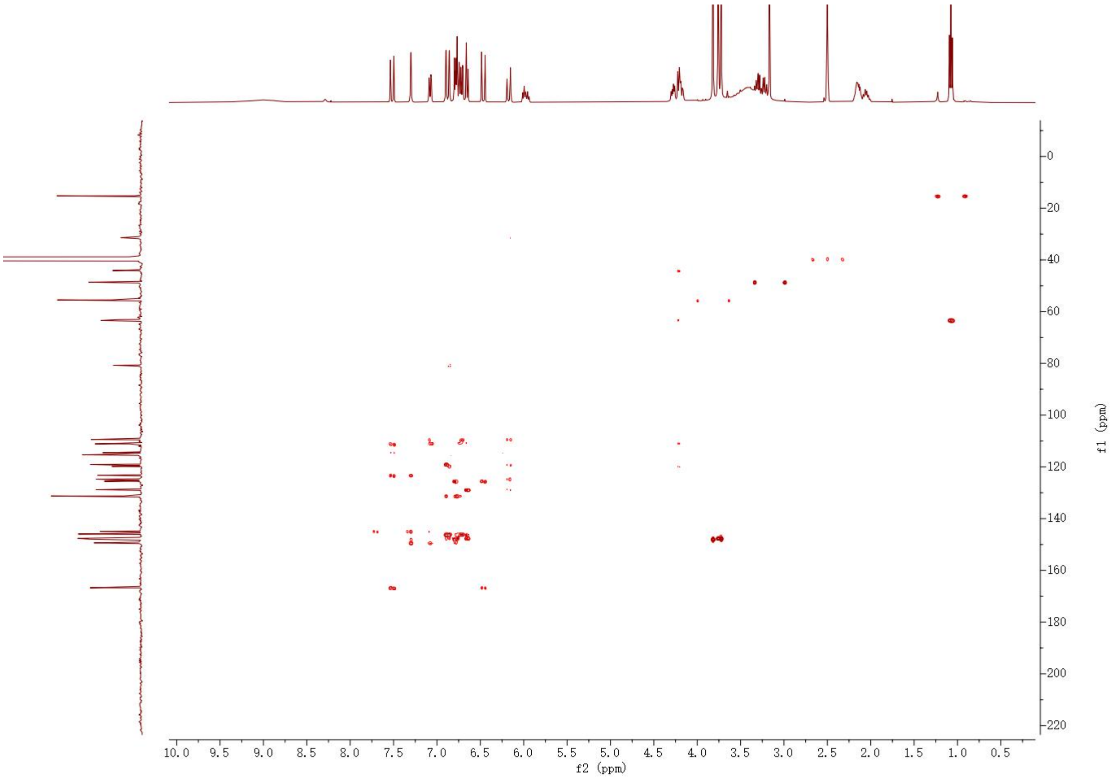

Figure S36. HMBC NMR spectrum (DMSO-d_6, 400 MHz, 100 MHz) of **2**.

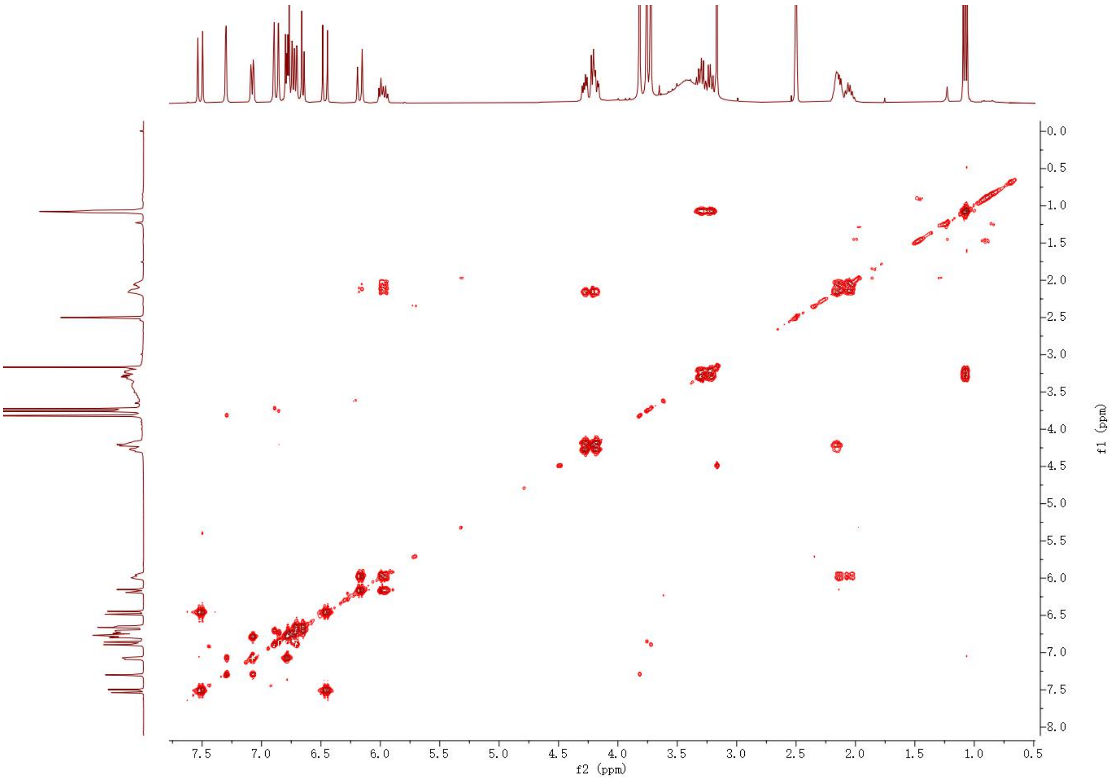

Figure S37. ^{1}H-^{1}H COSY NMR spectrum (DMSO-d_6, 400 MHz) of **2**.

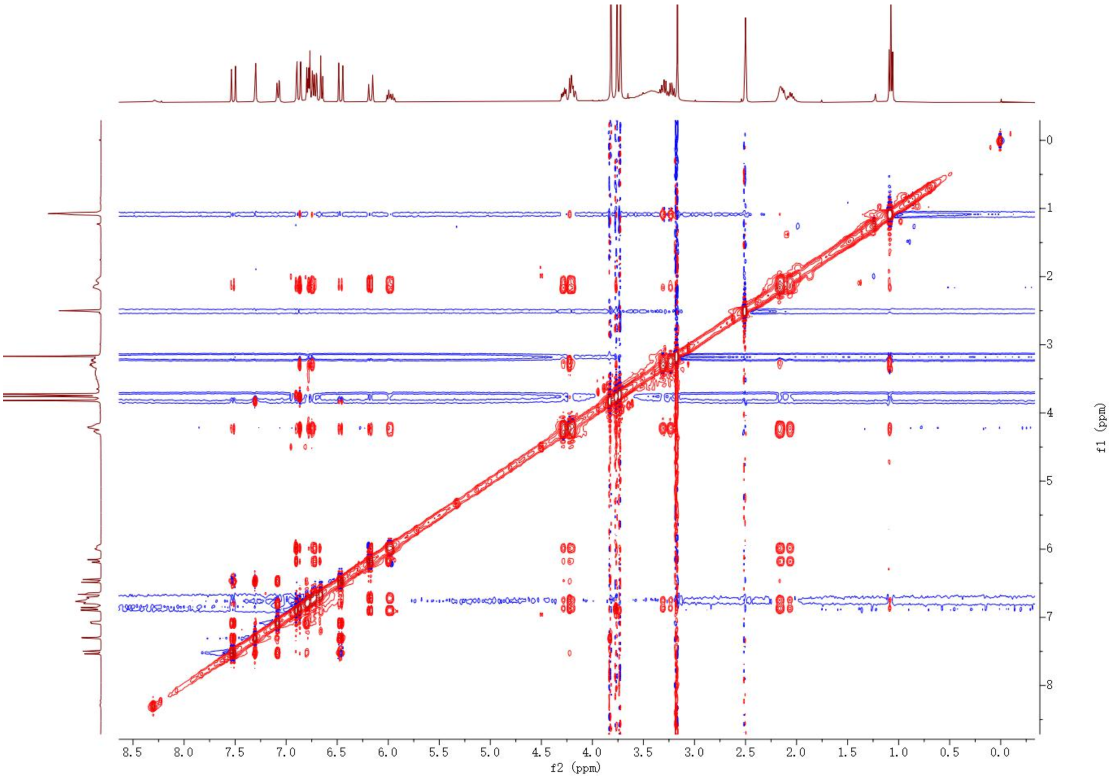

.Figure S38. NOESY NMR spectrum (DMSO-d_6, 400 MHz) of **2**.

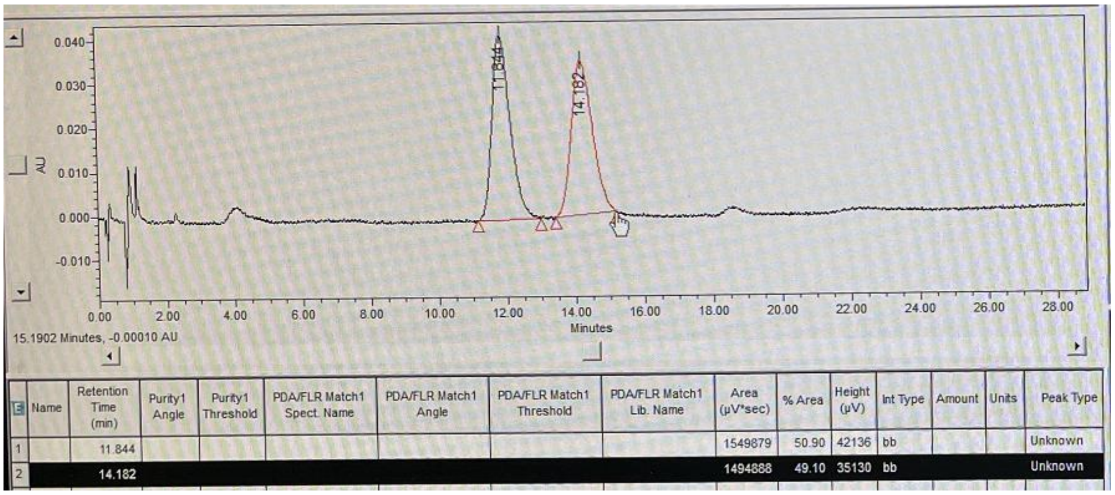

E	Name	Retention Time (min)	Purity1 Angle	Purity1 Threshold	PDA/FLR Match1 Spect. Name	PDA/FLR Match1 Angle	PDA/FLR Match1 Threshold	PDA/FLR Match1 Lib. Name	Area (µV*sec)	% Area	Height (µV)	Int Type	Amount	Units	Peak Type
1		11.844							1549879	50.90	42136	bb			Unknown
2		14.182							1494888	49.10	35130	bb			Unknown

Figure S39. Enantioseparation via chiral-phase UPC^2 on a Daicel Chiralpak IG column of **2**.
